# Quantitative Deep Sequencing Reveals Dynamic HIV-1 Escape and Large Population Shifts during CCR5 Antagonist Therapy *In Vivo*

**DOI:** 10.1371/journal.pone.0005683

**Published:** 2009-05-25

**Authors:** Athe M. N. Tsibris, Bette Korber, Ramy Arnaout, Carsten Russ, Chien-Chi Lo, Thomas Leitner, Brian Gaschen, James Theiler, Roger Paredes, Zhaohui Su, Michael D. Hughes, Roy M. Gulick, Wayne Greaves, Eoin Coakley, Charles Flexner, Chad Nusbaum, Daniel R. Kuritzkes

**Affiliations:** 1 Massachusetts General Hospital, Boston, Massachusetts, United States of America; 2 Harvard Medical School, Boston, Massachusetts, United States of America; 3 Los Alamos National Laboratories, Los Alamos, New Mexico, United States of America; 4 Santa Fe Institute, Santa Fe, New Mexico, United States of America; 5 Brigham and Women's Hospital, Boston, Massachusetts, United States of America; 6 Program for Evolutionary Dynamics, Harvard University, Cambridge, Massachusetts, United States of America; 7 Broad Institute of MIT and Harvard, Cambridge, Massachusetts, United States of America; 8 Fundacions irsiCaixa i Lluita contra la SIDA, Hospital Universitari Germans Trias i Pujol, Universitat Autònoma de Barcelona, Badalona, Catalonia, Spain; 9 Harvard School of Public Health, Boston, Massachusetts, United States of America; 10 Weill Medical College, Cornell University, New York, New York, United States of America; 11 Schering-Plough Research Institute, Kennilworth, New Jersey, United States of America; 12 Monogram Biosciences, South San Francisco, California, United States of America; 13 Johns Hopkins University, Baltimore, Maryland, United States of America; University of California San Francisco, United States of America

## Abstract

High-throughput sequencing platforms provide an approach for detecting rare HIV-1 variants and documenting more fully quasispecies diversity. We applied this technology to the V3 loop-coding region of *env* in samples collected from 4 chronically HIV-infected subjects in whom CCR5 antagonist (vicriviroc [VVC]) therapy failed. Between 25,000–140,000 amplified sequences were obtained per sample. Profound baseline V3 loop sequence heterogeneity existed; predicted CXCR4-using populations were identified in a largely CCR5-using population. The V3 loop forms associated with subsequent virologic failure, either through CXCR4 use or the emergence of high-level VVC resistance, were present as minor variants at 0.8–2.8% of baseline samples. Extreme, rapid shifts in population frequencies toward these forms occurred, and deep sequencing provided a detailed view of the rapid evolutionary impact of VVC selection. Greater V3 diversity was observed post-selection. This previously unreported degree of V3 loop sequence diversity has implications for viral pathogenesis, vaccine design, and the optimal use of HIV-1 CCR5 antagonists.

## Introduction

Infection with human immunodeficiency virus (HIV) is characterized by extensive viral diversity due to the high error rate of the reverse transcriptase, rapid viral turnover, and the impact of immune selection. Clonal analysis, single genome sequencing, and modeling provide evidence for the complex quasispecies nature of HIV-1 within infected individuals, but practical considerations have limited researchers' ability to document the true extent of viral heterogeneity. The advent of novel sequencing platforms that allow “deep” pyrosequencing of the HIV quasispecies provides an opportunity to confirm the previously hypothesized diversity of HIV-1 and to track the dynamic evolution of the quasispecies in response to a selection pressure.

Sequencing-by-synthesis technologies generate data by repetitive sequencing, or oversampling, of a given DNA segment and can be adapted to sequence one particular DNA region at great depth [1]–[3]. We used this approach to quantify and track diversity under drug selection pressure by sequencing V3 loop amplicons derived from plasma HIV-1 RNA of subjects receiving vicriviroc (VVC), an investigational CCR5 antagonist that inhibits HIV-1 entry [4]. The V3 loop of HIV-1 gp120 is the main determinant of viral cellular tropism, allowing the virus to use either the host cell surface proteins CCR5 (R5 viruses), CXCR4 (X4 viruses), or both (dual-tropic [D/M] viruses) as a coreceptor for entry [5]–[8]. CCR5 is used almost exclusively for entry in early infection, but CXCR4-using viruses associated with greater morbidity and mortality emerge in approximately 50% of patients over the course of infection [9]–[12]. The development of antiretrovirals targeting the gp120-CCR5 interaction has re-emphasized the need to improve our understanding of coreceptor usage [13]. In patients failing therapy with the CCR5 antagonist maraviroc, the dominant route of HIV escape was the emergence of CXCR4-using viral populations and not the development of conventional resistance [14], [15]. Algorithms to predict CXCR4 usage based on population sequencing of the V3 loop-coding region of *env* have low sensitivity for detecting X4 or D/M viruses in clinical samples [16]. For this reason, clinical trials of CCR5 antagonists have used a validated phenotypic assay to determine HIV-1 coreceptor usage and exclude patients with detectable CXCR4-using virus [17]. A phylogenetic analysis of viral sequences sampled from the time of maraviroc failure in two subjects suggested that CXCR4-using virus emerged on therapy from minor CXCR4-using viral populations that were not detected by the phenotypic assay [18]. New technologies that allow massively parallel sequencing of individual viruses in the HIV quasispecies could provide an improved representation of V3 diversity within a patient, and shed new light on the extent to which minor CXCR4-using populations and/or CCR5-using variants with reduced susceptibility to CCR5 antagonists circulate prior to CCR5 antagonist therapy [19].

## Results

### Validation of quantitative deep sequencing

Before we amplified and subjected patient samples to deep sequencing, we conducted duplicate control experiments to assess the effects of PCR amplification and deep sequencing with 454 technology on amplicon quantification and error rates using 3 clones from subject 07 at an input ratio of 89∶10∶1. The ratio was well preserved through the initial amplification and after post-processing filtering to exclude problematic sequences and to trim error-prone ends. These results indicated that no strong quantitative biases were introduced by the experimental or computational processing methods within the sensitivity of the control experiment (**Table S1**, **Figs. S1**, **S2**), confirming that quantification was reproducible for variants found at frequencies at least as low as 1%. After applying the filtering steps in the two controls, approximately 4.5% of sequences had one or more nucleotide differences from one of the three input sequences. Most of these (>99.8%) differed by only a single amino acid from one of the input sequences; the remainder (<0.2%) differed by more than one amino acid mismatch (**Tables S1 and S2**). Recombination was infrequently observed, but could only be clearly resolved when comparing the sequence present in the input 1% with the other two. The per-nucleotide error rate was 0.0011 and 0.0016 for the two control experiments, respectively, reflecting the error introduced by our combined amplification and deep sequencing protocol that remained after filtering out problematic sequences. For similar clones that differ by one nucleotide, our control experiments demonstrated a threshold of detection between 0.10–0.21%. We could not distinguish a true sequence difference from differences introduced by amplification errors or biases below this threshold. The threshold of detection necessarily decreased as the number of nucleotide differences increased.

To assess the reproducibility of sequence proportions determined by deep sequencing, we performed 4 replicate amplification reactions using identical input HIV-1 RNA (**Supplementary Methods S1**, **section 2.2**). The mean percentage (±SD) of a minor CXCR4-using variant obtained was 2.42±0.55%. The coefficient of variation was 22.8%, suggesting that for smaller fractions the absolute proportion measured by 454 sequencing can vary substantially around the true value.

### Subject selection, deep sequencing, data filtering and alignment

**Figure 1:**
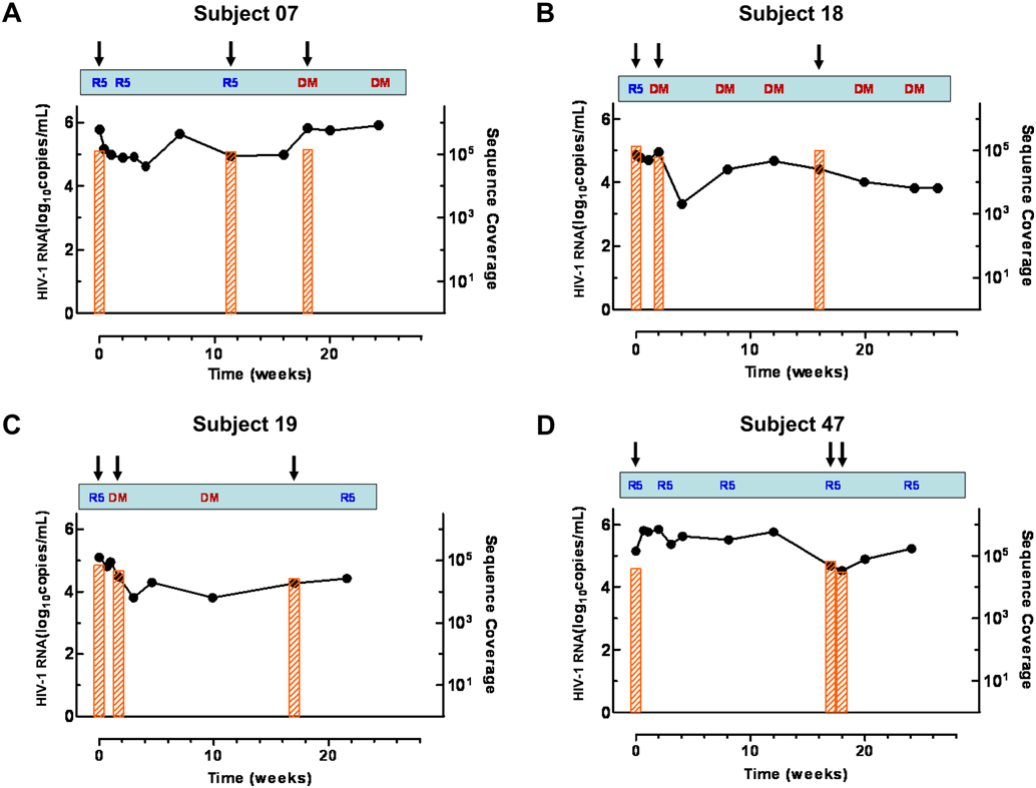
Plasma HIV-1 RNA levels, coreceptor usage and sequence coverage for four subjects failing VVC therapy. (A) Subject 07, (B) Subject 18, (C) Subject 19, (D) Subject 47. Protocol-defined virologic failure was defined as <1 log_10_ viral load decrease from baseline at or after week 16. Coreceptor usage was determined phenotypically by the Trofile assay; R5, CCR5-using virus only detected; DM, both CCR5- and CXCR4-using viruses detected. Vertical black arrows indicate time points sampled for V3 loop sequencing. The bars indicate the extent of coverage by deep sequencing.

We selected subjects enrolled in a phase IIb clinical trial of VVC who experienced protocol-defined virologic failure (<1 log_10_ viral load decrease at or after week 16) and a change in coreceptor usage as determined by a validated phenotypic assay (Trofile, Monogram Biosciences, South San Francisco, CA); subjects with assay-detectable CXCR4-using virus at baseline were excluded [13]. Per study protocol, all subjects received VVC for 2 weeks (baseline to week 2) prior to optimization of their background antiretroviral regimen. Three subjects met these criteria: subjects 18 and 19 had a rapid change to CXCR4-using virus whereas subject 07 developed high-level VVC resistance and had late emergence of a minority X4 population (**Fig. 1**). Subject 07 was infected with a subtype C virus, the others with subtype B; subtype C infections infrequently exhibit a CXCR4-using phenotype [20]. A fourth subject, subject 47, who had virologic failure without a change in coreceptor usage or VVC susceptibility served as a comparison. All subjects were receiving VVC at the time of virologic failure, but subject 19 had poor adherence and low VVC concentrations prior to virologic failure (data not shown). Three time points were analyzed for each subject: study entry (week 0), an intermediate time point while receiving VVC, and virologic failure. The first time point at which CXCR4-using virus was detected by the Trofile assay was selected as the intermediate time point for each subject; this occurred at week 2 for subjects 18 and 19. If CXCR4-using virus was not detected before virologic failure (subjects 07 and 47), we used the next time point after week 0 for which subject plasma was available as the intermediate time point. At baseline and week 2, genotypic susceptibility scores (GSS) were 0.0 and 0.37 for subject 07, 0.0 and 1.3 for subject 18, and 0.02 and 0.45 for subject 47, respectively (subject 19 did not have genotypic resistance data for either time point). Phenotypic susceptibility scores (PSS) at baseline and week 2 were 0.14 and 1.17 for subject 07, 0.0 and 1.88 for subject 18, 0.92 and 1.75 for subject 19, and 0.19 and 0.93 for subject 47, respectively.

Plasma HIV-1 RNA and subject-specific primer sets were used to reverse transcribe and amplify V3 loop-coding segments; amplicons were then sequenced in a blinded fashion using 454 technology [3]. Between 25,000–140,000 sequences were obtained per sample (**Table S3**). Real-time RT-PCR with SYBR green detection was performed for a subset of plasma samples to quantify the number of viral RNA templates being amplified with our primer sets (**Supplementary Methods S1**, **Section 1.3**). These data indicate that the proportion of templates amplified varied between 26–61% of the total templates available. Given the sequence heterogeneity within env, our use of subject-specific primer sets with degeneracy may represent a “best-case” scenario for template amplification; the use of primers based on consensus sequences could result in less efficient amplification.

Raw nucleotide sequences were filtered, aligned, trimmed and translated using pre-specified criteria applied uniformly so that all V3 sequence forms included in subsequent analyses spanned the complete V3 loop and the two proximal N-linked glycosylation sites. These filtering steps retained 80–95% of the original sequences for subsequent analyses, with the exception of one sample from subject 18 that had a higher proportion of sequences excluded due to a recurring frameshifting mutation (**Table S3**).

### Quasispecies V3 composition at baseline

**Figure 2:**
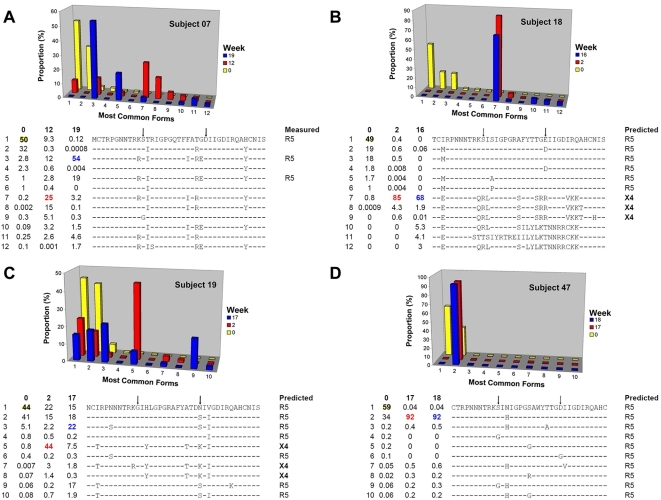
Longitudinal changes in V3 loop forms and proportions. (A) Subject 07, (B) Subject 18, (C) Subject 19, and (D) Subject 47. The most common V3 loop sequences across all three time points are numbered and displayed along the x-axis of the 3D-bar graph; corresponding amino acid sequences are shown below the graphs. The relative contribution of each sequence is plotted on the y-axis and displayed as a proportion of the total population. Time in weeks are shown on the z-axis. A coreceptor usage prediction using PSSM is shown for each sequence [32]. Coreceptor usage was measured phenotypically in sub07 by generating recombinant viruses that incorporated each V3 loop sequence. Vertical arrows denote positions 11 and 25 in the V3 loop, respectively.

Baseline samples demonstrated considerable V3 loop sequence heterogeneity. In all subjects, about half (44–59%) of the baseline viral population consisted of a single dominant R5 V3 form; the three most common baseline forms accounted for the majority (86–93%) of the population in each sample (**Fig. 2**). The remainder of each viral population consisted of hundreds or thousands of low frequency minor forms, even over the very short region of the V3 loop (117 bases, or 39 amino acids) that was the focus of the study. Subjects 18 and 19 had a number of forms that were predicted and/or phenotypically confirmed to be X4, (**Figs. 2b,c**, **Tables S4**, **S5**) showing that rare X4 sequences can co-exist within a dominant R5 population at levels undetectable by conventional methods. Because subject 07 was infected with an HIV-1 subtype C virus, accurate prediction of CXCR4 usage by genotype was precluded. However, phenotypic testing (Trofile) of the uncloned plasma virus population indicated exclusive CCR5 use at the time points studied with 454 sequencing (**Figure 2a**). Subject 47, who did not exhibit a coreceptor change, had very few predicted baseline X4 V3 forms and, as will be described, relatively restricted V3 diversity (**Figure 2d** **and Table S6**).

### Rapid selection of pre-existing minor V3 loop variants under VVC pressure

By week 2 of VVC treatment, frequencies of the dominant R5 V3 forms in subjects 18 and 19 fell, while those of select minor CXCR4-using forms rose dramatically (**Figs. 2b,c**). In subject 18, the three most common baseline forms declined from a total of 86% to just 1.5% of the V3 loop population, while a CXCR4-using form present at 0.8% of baseline sequences rose to 85%. In subject 19, the three most common baseline forms fell from a combined prevalence of 90% to 38%, while an X4 form that was again present at 0.8% at baseline rose to become the most common form (44%) at week 2. By week 12, subject 07 showed a similar pattern. The three most common baseline forms, which constituted 85% of the population declined to 22%, while three forms that were present at a combined prevalence of 3% at baseline rose to 52% of the population. No CXCR4-using virus was detected, however, and the forms that increased in prevalence during VVC treatment are known from earlier work to confer VVC resistance [21]. Subject 47, in whom two R5 forms that differed at a single amino acid position were present at baseline (N308 vs. H308, 59 and 34% respectively), had a switch in dominant forms without the emergence of CXCR4-using virus or VVC resistance (**Fig. 2d**). The H308-containing form rose to 92% at weeks 18 and 19, suggesting a relative fitness advantage of this variant in the presence of VVC. In every case, the forms that were dominant at the later time points were present as minor variants in the baseline populations.

A subset of CXCR4-using forms that rose to plurality in subject 18 during VVC treatment persisted through week 16. In subject 19, self-reported non-adherence and low plasma VVC levels at week 8 were accompanied by a resurgence of the three most common CCR5-using baseline forms (**Fig. 2c**). The CXCR4-using form that was most common while the subject remained adherent fell 6-fold from 44% at week 2 to 7.5% at week 17.

### Evolutionary impact of VVC selection

**Figure 3:**
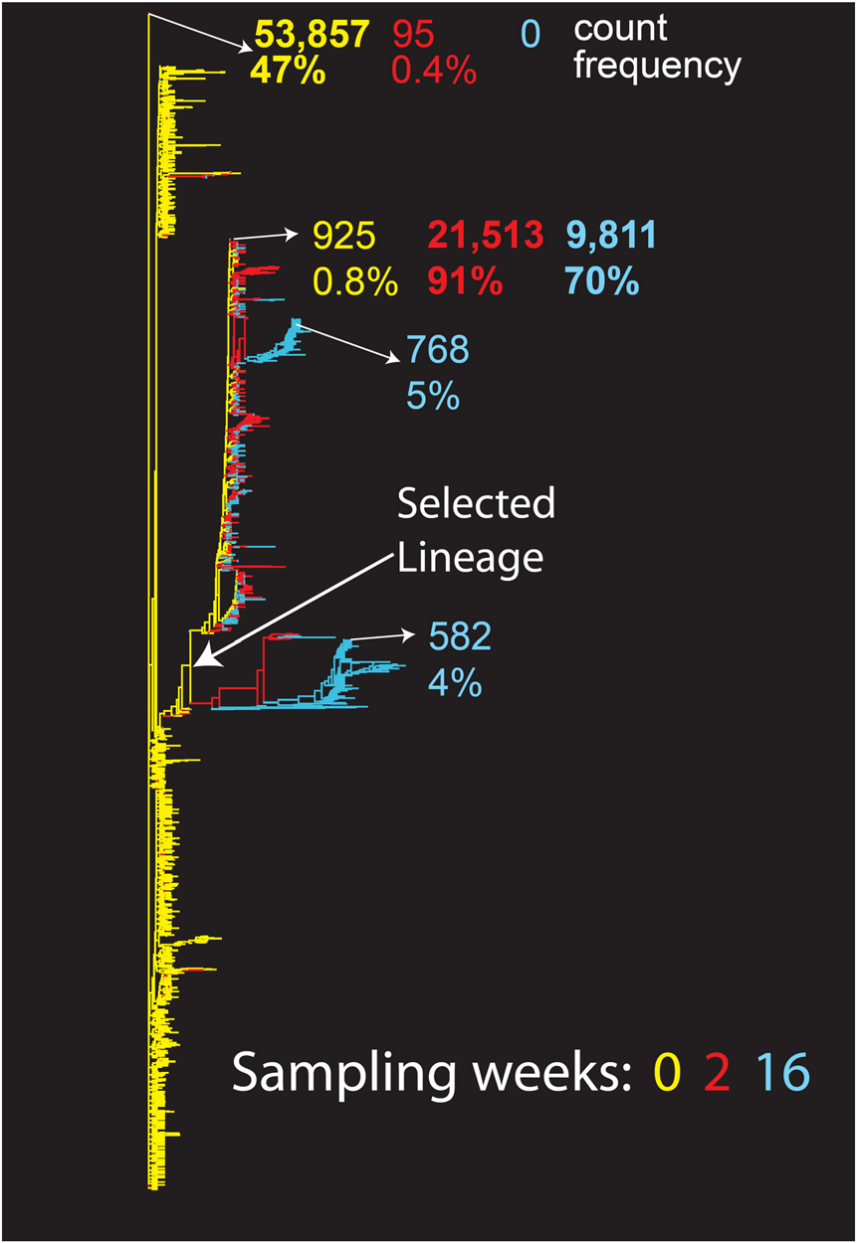
Neighbor-joining (NJ) tree over time for subject 18. All unique V3 forms found are included, and the frequency of the 3 most common nucleotide forms indicated at each time point. The most common sequence at the first time point was used as an out-group for the trees. Week 0, yellow; week 2, red; week 16 (virologic failure), blue. We have indicated the location and frequency of the most common sequence at each time point, and the predominant lineage after selection.

**Figure 3** shows a neighbor joining (NJ) tree of all unique forms found in subject 18, selected as a representative case; the NJ trees of the other 3 cases are provided in the supplement (**Figure S3**). This tree illustrates both the clear evolutionary trajectory of the virus over 16 weeks of sampling, and the complexity of the V3 loop at baseline. There were 1,910 unique variants in this 39-amino acid fragment of the virus in these samples. A remarkable diversity of forms was already present in the baseline sample. The majority of these forms are expected to represent true biological variants, rather than PCR or sequencing artifact, given the relatively small fraction of experimental error that remained in the sequences after processing (see above discussion and compare **Fig 3** with the control tree shown in **Fig S2**). The most common form of the V3 loop at week 0 was found in only 0.4% of the sequences at week 2, and was lost by week 16. There was significant selection for a single lineage at the second time point (week 2), with one X4 sequence from that lineage making up 91% of the sample; this form of the V3 loop continued to be the most common form at the third time point (week 16), persisting at 70%. Interestingly, despite a genetic bottleneck imposed by VVC, many minor related variants within the selected lineage persisted at low levels and continued to evolve alongside the dominant X4 form. This point can be visualized in the tree by the acquisition and building of new variants from the many variants present at week 0 within the selected lineage, and by the left to right progression from week 0 to 2 to 16 (yellow to red to blue) within that lineage. Two distinctive novel lineages were detected at week 16, constituting 4% and 5% of the sample, respectively. The branch lengths after VVC selection are relatively long compared to relationships among baseline sequences. These findings suggest that rather than rapidly replacing the susceptible form of the virus with a single X4 variant, new levels of diversity in the V3 loop were explored under the selective pressure of VVC. Many variants were carried forward and continuing evolutionary pressure drove de novo exploration of the sequence space.

Comparable sequence phylogenies for subjects 7 and 19 share interesting features with subject 18 (**Fig S3**, **S4**, **S5**). All three show extreme baseline diversity, selection of a favored lineage at the second time point (rather than a single escape variant), and the emergence under VVC selection of distinctive lineages with relatively long branch lengths compared to the baseline sequences. The emergence of novel forms that evolved from the population suggests that the virus may have the opportunity to explore previously unvisited sectors of sequence space after drug pressure selects against the previously dominant baseline form(s). In subject 19, non-adherence to the VVC regimen was associated not with reversion to the most common baseline sequence, but with outgrowth of one of the other major lineages present at baseline (**Fig 2c** and **Fig S3**, **S4**, **S5**). Virus from subject 47, in whom virologic failure was not associated with emergence of VVC resistance or CXCR4-using variants, had much lower diversity overall over time.

**Figure 4:**
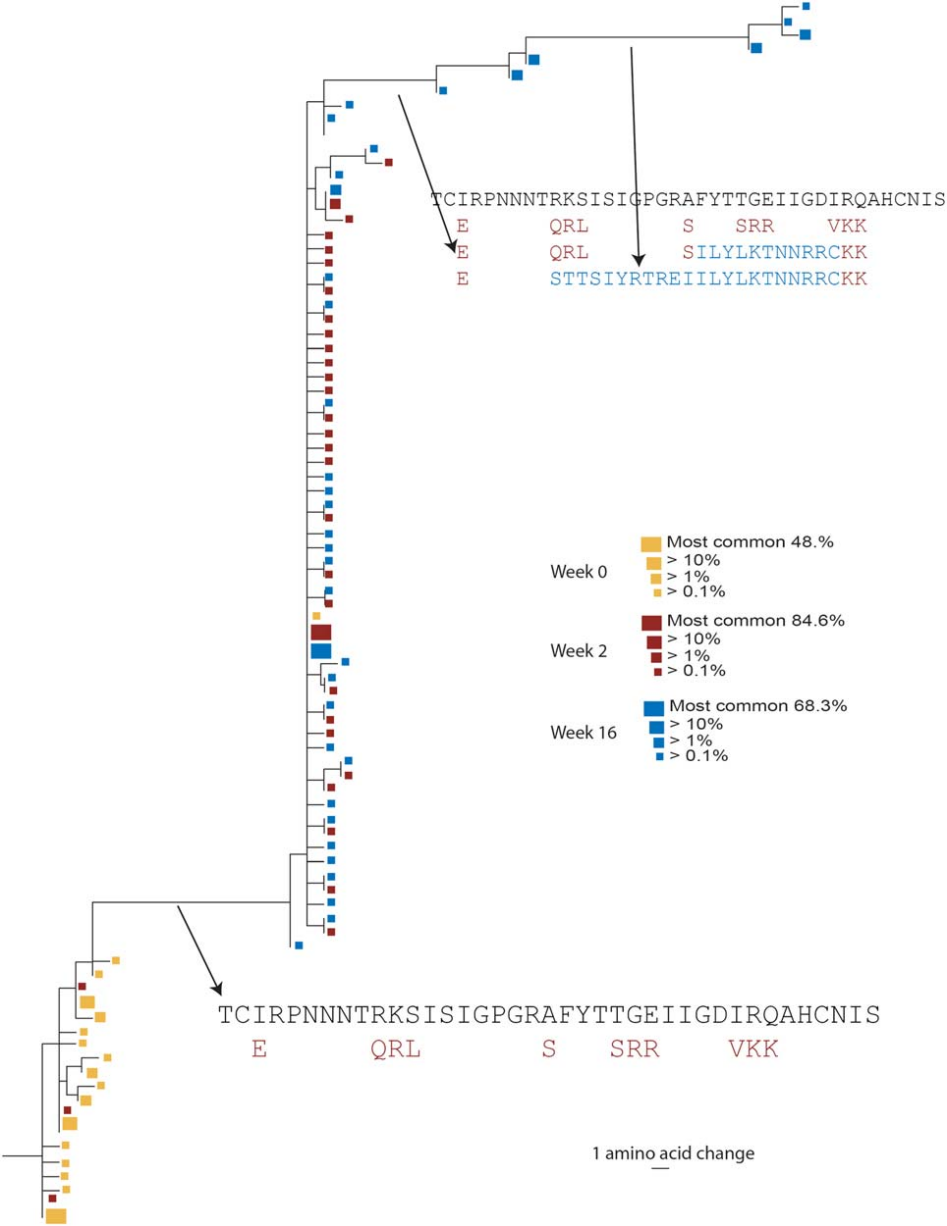
Maximum Likelihood (ML) tree of the unique V3 forms for subject 18. These trees include only sequences found in more than 0.1% of the total. Amino acid sequences at branch points are shown. Week 0, yellow; week 2, red; week 16 (virologic failure), blue. The size of the box at each leaf node indicates the frequency of the occurrence of the sequence.

The NJ trees enable an overview of each of the unique forms of the virus and their relationships, but these trees do not capture the rapidly shifting frequencies of the viral forms—the most striking manifestation of the VVC selection—in the context of their evolutionary trajectory. Thus, maximum likelihood (ML) trees including only sequences found at frequencies greater than 0.1% of the populations are also provided. These trees label unique sequences by the magnitude of their population frequency. Again, subject 18 is used as the example in **Fig 4**; ML trees for the control experiments and for the remaining 3 subjects are provided in the supplement (**Fig S1 and S4**, respectively). As each sequence included in this analysis is repeated many times in a sample, all variants included in the ML tree are likely to be found in vivo. For the ML trees, the key amino acid sequences that typify emerging clades are shown. The most common baseline sequence was used as an outgroup for both the NJ and ML trees, allowing visualization of population shifts and the acquisition of new viral forms building off of the spectrum of baseline variants, with a clear progression over time (**Figs. 3**, **4**, **S3**, **S4**, **S5**).

Very unusual forms of the V3 loops, including some that do not have an obvious conserved GPG turn at the tip, were found at the third time point in subject 18 (**Fig 4**). These forms were primarily the result of compensatory frameshifts within this short region. As part of our processing strategy, we excluded all sequences with frame-shift errors that are not compensated for, but we retained frameshifts with paired nearby compensatory insertion-deletion patterns, as HIV frequently uses compensatory frame-shifting as a means of adaption [22]. Single base insertions or deletions are a common experimental error in 454, however, particularly in homopolymer reads due to the non-linearity of the signal [23]. Therefore, compensatory paired 454 errors could be a trivial explanation of these odd V3 sequences. The particular array of frame-shifting mutations that gave rise to the two clusters observed in subject 18, however, are not all embedded in strings of like bases. It is possible that the unusual V3 sequences may be viable despite their very distinctive form, as they do not represent common 454 errors in the earlier points from this subject, but are common only in the third sample after 16 weeks under selective pressure. In addition, these sequences represent clusters of variants suggestive of evolutionary progression. Thus the source of the unusual pattern–experiment error or biological variation– remains unresolved. The long branch-lengths reflect alignments optimized to provide intact reading frames and are not representative of base changes, and so they reflect the divergence of the translated loop at the amino acid level.

Deep sequencing also enabled tracking of temporal changes in the proportions of the predicted minority X4 V3 forms that were present at baseline in study subjects (**Tables S4**, **S5**, **S6**). Here, patterns were mixed. Some forms increased in frequency, while others decreased or disappeared.

### Changes in Shannon Entropy over Time

**Figure 5:**
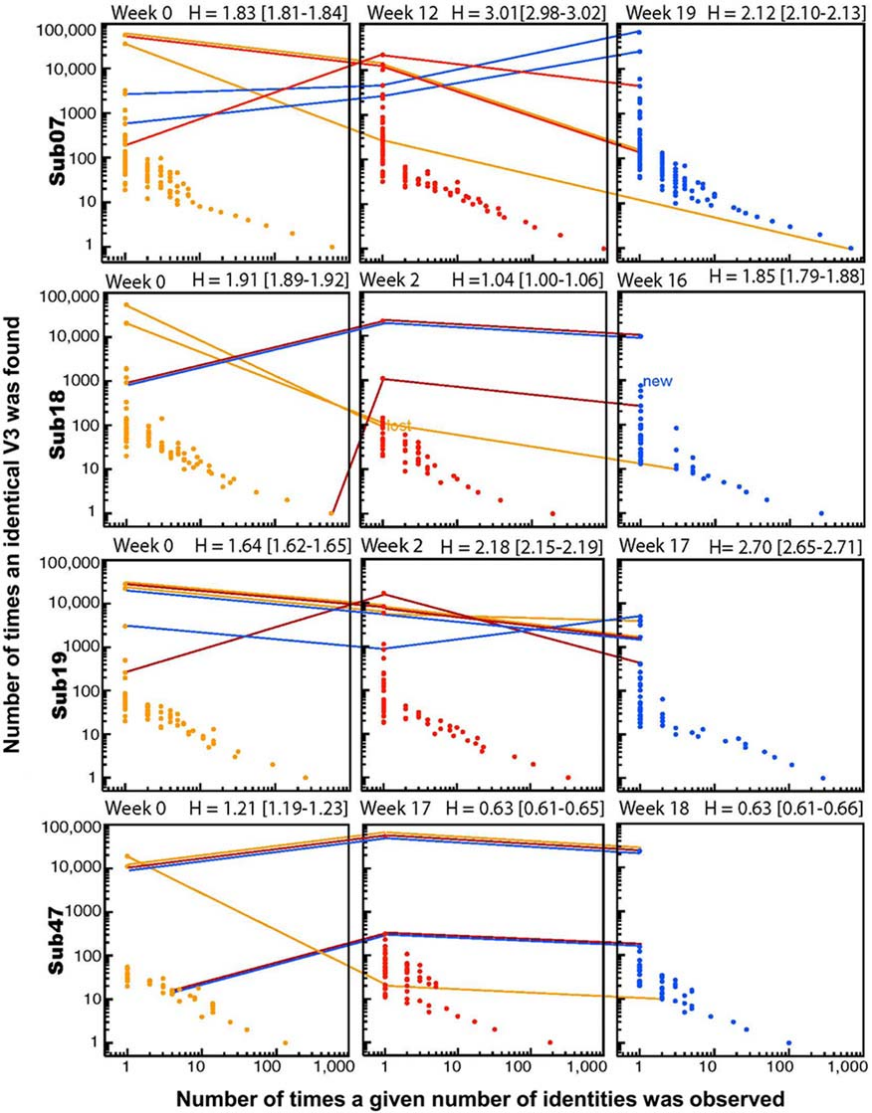
Shannon Entropy Plots. The number of times each form is found is plotted against the number of forms found with a given frequency. Week 0, yellow; intermediate time point, red; VF, blue. 95% CI for the Shannon Entropy are calculated by 100 bootstrap re-samples of the distribution of forms at each time point. The rise and decline of the two most common forms at each time point are tracked through the figures and are represented by the colored lines; the coloring of these lines facilitates tracking the most common forms at the first time point (yellow), second time point (red), and third time point (blue) through the time sequence.

Each unique V3 form at each time point was considered, and the number of times each form appears was used to calculate the Shannon entropy of the sample (**Fig 5**). In this analysis the V3 loop is being treated as a functional unit, and every distinct form considered independently of distance measures, to obtain a different perspective regarding population complexity. The 95% confidence intervals (CI) for the Shannon Entropy were calculated by 100 random-with-replacement re-samples (bootstrap) of the primary V3 loop data at each time point. To illustrate the sample diversity for each point, we plotted the number of times each form was found on the y-axis, by the number of forms found with given frequency on the x-axis. Thus, at week 0 for subject 07 there were 112,818 V3 sequences that were intact with no frame shifts or stop codons (N = 112,818). The most common sequence was found 56,546 times (the top left hand point). There were 585 unique sequences that might be viable as they were intact in the V3 region, but they were each found only once in the sample (gold point on the bottom right). The Shannon entropy, H, was 1.83 [95% CI 1.81–1.84]; the 95% CI of the entropy for this time point and the next did not overlap, so the second sample was significantly more diverse. After VVC selection, the total V3 diversity increased in each subject with emergent X4 or VVC-resistant virus, corresponding to a significant increase in the overall sample Shannon entropy in subjects 07 and 19. The seeming paradox of increasing entropy of V3 forms upon selection in these cases suggests that the resistant viruses may have the opportunity to explore sequence space and variants can compete more effectively after drug pressure selects against the previously dominant and fit baseline form(s).

## Discussion

Deep sequencing affords multiple advantages over current sequencing techniques for the detection of minority HIV variants. Co-linear sequence can be obtained in areas of significant genetic heterogeneity, characteristic of HIV *env*, and at a faster speed and greater depth than conventional cloning or single genome sequencing. We developed a rigorous methodology for the filtering and validation of quantitative deep sequencing in the context of viral infections that can readily be applied to other deep sequence data sets. Quantitative deep sequencing of plasma samples from subjects failing a CCR5 antagonist-containing regimen provided orders of magnitude greater coverage than previously possible and revealed that minor V3 loop sequence variants have significant clinical implications in chronic HIV-1 infection. We directly demonstrated the previously hypothesized vast diversity of intra-patient HIV sequences, in this case within a variable determinant of viral coreceptor usage, and quantified the dynamic sequence variation over time [24].

Although variants using either CXCR4 or CCR5 are known to coexist in plasma, deep sequencing detected a multitude of distinct co-circulating V3 forms and highlighted the extraordinary diversity of V3 sequences available to the virus as it adapted to changing fitness environments. Variants with predicted CXCR4 usage as well as VVC-resistant variants that emerged during VVC treatment were shown to exist as uncommon or rare forms in baseline samples in three of the four subjects we studied. These rare forms differ from the dominant V3 form at multiple amino acid positions and exist at greater frequencies than the single cycle mutation rate of HIV (10^−5^–10^−4^ per base per cycle) or the observed in vivo frequencies of non-nucleoside reverse transcriptase or protease inhibitor resistance mutations (0.03% and 0.03%, respectively) in treatment-naïve HIV-infected patients [24]–[27]. This finding suggests that these V3 loops are not generated and lost with each replication cycle, but in fact may be maintained as minority variants during chronic infection. The documentation of extensive V3 sequence diversity, coupled with the rapid expansion of rare variants in response to a drug selection pressure highlights the extraordinary challenge HIV diversity poses for preventive or therapeutic vaccines targeting areas of similar sequence heterogeneity. These data further provide compelling evidence that minority HIV variants present at less than 1% of the HIV-1 population in plasma are clinically relevant. The level of a minority variant that is clinically relevant most likely varies as a function of the potency of the background antiretroviral regimen, underscoring the importance of combining several potent agents when constructing antiretroviral regimens.

The reason subject 47 experienced treatment failure remains unclear. The low GSS and PSS of this subject's antiretroviral regimen, determined at baseline and week 2, suggest that limited potency of the antiretroviral regimen was the most likely explanation for failure to achieve or maintain a 1-log_10_ reduction in viral load. Subject adherence and plasma VVC levels were adequate, and no changes were observed in coreceptor tropism, phenotypically or genetically. Although most mutations that confer CCR5 antagonist resistance map to the V3 loop, sequence changes in other regions of *env* have been noted (M Lewis, et al, 15^th^ Conference on Retroviruses and Opportunistic Infections, February 5–8, 2008, Boston, MA, Abstract 817) [21], [28], [29]. It is possible that sequence changes in env outside of the V3 loop could have contributed to virologic failure, but phenotypic testing with full-length *env* constructs from subject 47 did not demonstrate any loss of VVC susceptibility.

Our analysis has important limitations. Some error is inevitably introduced through these experimental methods and it is not obvious precisely which of the rare forms are the consequence of this sequencing error. Furthermore, the longitudinal nature of the data allowed tracking of frequency shifts in interesting sequences that were subject to VVC selection pressure. Because of the limited length of the sequences, homoplasy rather than shared lineages may underlie some of the observed clustering patterns in the trees, and in vitro recombination during amplification may also have contributed to some of the apparent clades [30]. The trees are not expected to provide accurate renditions of the phylogenies as they are by necessity constrained by limitations imposed by the nature of the data (very short sequences, convergence in a scenario of emerging drug resistance, recombination, critical mutations occurring by insertion and deletion), and are based on thousands of short fragments with some experimental error. However, they do capture the complexity of the samples, as well as the rapid temporal shifts in the evolutionary landscape as a consequence of selective pressure. Clear evolutionary progression over time was evident in each subject. Multiple co-circulating minor CXCR4-using viruses were found to exist in chronic infection, and the virus simultaneously explored many escape routes as the drug-susceptible form was selected against. The process of escape may push the virus towards sampling unexplored regions of the sequence space. Taken together, our data demonstrate the feasibility and utility of harnessing deep sequencing platforms to comprehensively assess viral diversity, quantify minor sequence variants, and provide insight into the mechanisms of viral escape from novel CCR5 antagonists.

## Methods

### Subject Selection

We selected subjects enrolled in a phase IIb clinical trial of VVC who experienced protocol-defined virologic failure and a change in coreceptor usage as determined by a validated phenotypic assay (Trofile, Monogram Biosciences, South San Francisco, CA), excluding subjects with assay-detectable CXCR4-using virus at baseline [13]. Plasma samples were required to have HIV-1 RNA levels ≥5,000 copies/mL at all time points analyzed. Genotypic and phenotypic susceptibility scores were calculated at baseline (week 0) and week 2.

### RNA Preparation

Virus from 500–1,400 µL of plasma was pelleted by centrifugation (17,000×*g*, 1 hr, 4°C), resuspended in 140 µL of plasma, and extracted using the QIAamp viral RNA mini kit according to the manufacturer's instructions (Qiagen, Valencia, CA; see **Supplementary Methods S1** for sample-specific plasma volumes). The entire derived volume of extracted RNA (60 µL) was used for cDNA synthesis. The range of the maximum RNA copy number extracted from clinical samples was 18,000–793,000 (as quantified by the HIV-1 Monitor assay [Roche Molecular Systems, Pleasanton, CA]), depending on available plasma volume and total viral load in a given sample. The actual number of viral templates amplified with our protocol was determined for three samples (see Template Number Validation below).

### Primer Design

Given the heterogeneity of envelope sequences in the region flanking the V3 loop, subject-specific primers were designed for cDNA synthesis and DNA amplification. Ten to 20 full-length *env* clones were isolated from plasma samples obtained at study entry and at virologic failure. These full-length clones were used to assess sequence heterogeneity and to guide appropriate design and placement of forward and reverse primers. Despite the limitations on amplicon size imposed by 454 sequencing, areas of no or limited sequence heterogeneity that were suitable for primer design could be identified in the regions immediately proximal and distal to the V3-coding sequence. Primer degeneracy was introduced when polymorphic positions in the target sequence could not be avoided. The resulting primer sets amplified the entire V3 loop-coding region of *env* with approximately 50–75 additional nucleotides of flanking sequence. (Specific primers used were as follows: subject 07 1^st^ round 5′-RCCAGTGGTRTCAACTCAAC-3′ (07.1f) and 5′-CCTRMGGRTGGTTGAAAAC-3′ (07.1r), 2^nd^ round 5′-GGTAGCCTAGCRGAAGGRAAG-3′ (07.2f) and 5′-CATTCCATTGCYTTKCACTA-3′ (07.2r); subject 18 1^st^ round 5′-GCCAGTAGTATCAACTCAAC-3′ (18.1f) and 5′-CAATTTCTGGGTCCCCTCCTG-3′ (18.1r), 2^nd^ round 5′-GAAGGAACCTGTAAATATTAC-3′ (18.2f) and 5′-TCCAKTCTGYTKYACTAATG-3′ (18.2r); subject 19 1^st^ round 5′-GGCAGTCTAGCAGARGAAGAGG-3′ (19.1f) and 5′-CCTGAGGATTGMTTAAAGGC-3′ (19.1r), 2^nd^ round 5′-CARCTGAATGAATCTGTAAC-3′ (19.2f) and 5′-CCAGCTTKTTYCACTAATGTTAC-3′ (19.2r); subject 47 1^st^ round 5′-GGCAGTYTAGCAGAAGATGAGG-3′ (47.1f) and 5′-GGTCCCCYCCTGAGGATTGG-3′ (47.1r), 2^nd^ round 5′-GATCTGAGAATTTCACAAACAATGC-3′ (47.2f) and 5′-TCCATGTTGCTCTACTAATG-3′ (47.2r).

### Conventional RT-PCR and PCR

The extracted RNA (60 µL) was added to the RT-PCR reaction mix (final volume, 200 µL) that contained: 1× RT buffer, forward and reverse primers (200 nM each), dNTPs (200 µM), MgSO_4_ (1.7 mM), and SuperScript III RT/Platinum *Taq* enzyme mix (Invitrogen, Carlsbad, CA). The reaction was transferred to thin-walled PCR tubes (USA Scientific, Ocala, FL) in 50 µL aliquots and subjected to the following conditions: 55°C for 30 minutes, 94°C for 2 minutes, and 30 cycles of 94°C for 30 seconds, (T)°C for 30 seconds, and 68°C for 20 seconds (where T equals 5°C below the lowest primer T_m_). A final extension was performed at 68°C for 5 minutes, after which the aliquots were pooled into a 1.7 mL centrifuge tube, purified (MinElute PCR Purification Kit, Qiagen), eluted into 30 µL of RNAse/DNAse-free water, and used as template for a second round of amplification. The entire 30 µL of first round PCR product was added to a PCR reaction (final volume 200 µL) that contained: 1× high fidelity buffer, forward and reverse primers (200 nm each), dNTPs (200 µM), MgSO_4_ (2 mM), and 4 units Platinum *Taq* High Fidelity enzyme (Invitrogen, Carlsbad, CA). This reaction mixture was then apportioned into 50 µL aliquots for thermocycling under the following conditions: 94°C for 2 minutes, then 30 cycles of 94°C for 30 seconds, (T)°C for 30 seconds and 68°C for 20 seconds (where T equals 5°C below the lowest primer T_m_) . After amplification, the PCR products were purified as above prior to 454 sequencing.

### Template Number Validation

The number of viral templates that were amplified by our protocol was determined experimentally for a subset of plasma samples. Using subject-specific primer sets, we used a real-time RT-PCR assay with SYBR green detection to quantify RNA templates from Subject 18 baseline and Subject 47 baseline and week 16 plasma samples. Based on results of the HIV-1 Monitor assays, a plasma volume corresponding to 56,828 RNA copies from subject 18 baseline plasma, 196,425 copies from baseline subject 47 plasma, and 47,108 copies of subject 47 week 16 plasma were centrifuged at 17,000×*g* for 1 hour at 4°C. The pellet was resuspended in 140 µL of plasma and extracted into 40 µL of buffer AVE using the QIAamp Viral RNA Mini Kit according to manufacturer's instructions (Qiagen). Ten microliters of this RNA was used in a real-time PCR reaction that contained 1× Power SYBR Green RT-PCR Mix, 1× RT Enzyme Mix, 200 nM of forward and reverse primer, either 18.2 f/r or 47.2 f/r, in RNAse-free water to a total volume of 50 µL (Power SYBR Green RNA-to-Ct 1-Step Kit, Applied Biosystems). All samples were run in duplicate. Each assay run contained two negative controls (a no template control and a no reverse transcriptase control) and a standard dilution curve. The standard curve with a dilution range of 10^2^–10^7^ copies/mL was generated using RNA synthesized from a plasmid that contained the dominant subject-specific V3 loop sequence from the baseline (week 0) sample (TOPO-TA Kit, [Invitrogen] and Megascript T7 High Yield Transcription Kit [Ambion]). RNA was quantified spectrophotometrically, diluted, aliquoted, and stored at −80°C; aliquots of RNA standards were thawed only once before use. Real-time PCRs were performed using an ABI Prism 7000 Sequence Detection System (Applied Biosystems) for one cycle of 42°C for 40 minutes, one cycle of 95°C for 10 minutes and 45 cycles of 95°C for 15 sec and 55°C for 1 minute. The standard curves generated for subjects 18 and 47 had an r^2^ value of 0.974 and 0.995, respectively. The number of amplified viral templates from subject plasma samples was estimated by comparing the sample cycle threshold to the threshold values for the known RNA copy numbers of the standard curve (**Supplementary Methods S1**, **Section 1.3**).

### Deep Sequencing, Data Filtering and Alignment

The V3 loop amplicons were first end-repaired with T4 DNA polymerase and 5′-phosphorylated with T4 Polynucleotide Kinase (454/Roche). Amplicons were then column purified (MinElute, Qiagen). Standard 454 adapters (one of which was biotinylated) were then ligated to the amplicons using DNA ligase (454/Roche). The modified amplicons including adapters were column purified (MinElute, Qiagen), immobilized onto 28 µm streptavidin beads, and blunt ended (454 adapters have a 5′ overhang) using a Fill-in polymerase (454/Roche). The single-stranded 454 library was then isolated as described and quantified using an Agilent 2100 Bioanalyzer [3]. The 454 libraries were amplified on beads with emulsion PCR and pyrosequenced as described [3]. All ultradeep sequencing was performed using 454 GS FLX sequencing platforms (Roche, Palo Alto, CA) at the Broad Institute at Harvard and MIT. The raw data were processed, filtered, and reported as readable sequence. Quality scores were derived using predictors that improved on the default system software [31]. For subject plasma samples, deep sequencing coverage (i.e., the number of unique sequences generated) approximated the maximum input viral RNA copy number. The depth of sequence reads therefore varied from sample to sample.

### V3 Loop Sequence Alignment Strategy

Because of the jagged start and stop positions and multiple frameshifts and duplications, the standard multiple alignment tools we used failed to give useful alignments of the 454 ultra deep sequence nucleotide data. We therefore developed a series of computational tools that align deep sequencing datasets for subsequent analysis and interpretation. We first created an alignment by hand from one sample to determine the type of problem sequences we would encounter during the alignment process. Sequences were generated in both the forward direction and as reverse complements. Some were fragments that did not span the entire V3 loop. Many were frame-shifted, with one- or two-base insertions or deletions; there were frameshifting mutations between almost every codon somewhere in the full alignment in these very large data sets. Finally, some sequences contained imperfect direct repeats, where the entire 454 sequence or a large fraction of it was repeated. We did not know if a particular error was due to HIV reverse transcriptase (RT) or was introduced during the experimental procedure, but all the errors described above would result in a non-viable virus, and so we excluded them using an algorithm to generate a working V3 amino acid alignment. The regions near the end of the sequences tended to by the most error prone, so sequences were trimmed to the 117 bases encompassing the V3 loop and proximal glycosylation sites for subsequent analyses.

Our filtering and alignment process began by compressing all identical sequences into sets such that a single representative sequence was included, and named to indicate how many times it was found in the sample. These sequences were rank-ordered from most to least common and sequentially numbered to give every sequence a unique name. This compression step was repeated several times through the filtering process – to the initial raw DNA sequences, to the aligned DNA sequences trimmed to the region of interest, and to the final protein alignment. We then generated a reverse complement sequence from the entire set, and by aligning each sequence in a pair-wise alignment to HXB2, checked the similarity so that we kept only the forward direction version of the sequence. Essentially any HIV strain can serve this purpose; HXB2 was used for convenience, as it is a standard reference strain. The goal was to confirm the forward or reverse direction of the read and screen out aberrant sequences. Sequences that were <60–70% similar to HXB2 even in the forward direction were also excluded (this threshold was tuned so that we didn't lose good sequences in the more distant subtype C samples of subject 07; within-patient consensus sequences can be also used with a higher similarity threshold). Spot-checking of the sequences excluded due to low similarity indicated that they generally comprised sequences that contained relatively long (given the length of the sequence) direct repeats. We then eliminated all sequences that were too short to span our minimum region of interest - the V3 and the two proximal glycosylation sites (39 amino acids = 117 bases).

We next created pair-wise alignments of each sequence to HXB2, made a full DNA alignment by sequentially combining the pair-wise alignments, codon aligned the multiple sequence alignment using HXB2 coding information (using the strategy we developed for the GeneCutter tool at the Los Alamos database [www.hiv.lanl.gov]). At this step, we segregated any sequences containing a frame shift (single or double base gaps or insertions) within the V3 loop. Sequences that had nearby compensatory changes were retained in the working alignment. Then we translated all sequences and recompressed the file to rename it according to the number of protein sequence identities. All sequences with a stop codon were removed, as well as a small number of sequences that did not fully span the V3 loop that were not successfully filtered out. The resulting protein alignment was reviewed and finalized using the multiple alignment sequence editor MASE.

**Table S3** shows how many sequences we eliminated at each step of the filtering process. Sequences from subject 18 had a recurrent frame shift in the middle of the V3 loop in a string of 6 A's, a known common error in the 454 sequencing process– it was the most common form in the week 02 and week 16 samples from this subject, and is why such drastic cuts were made in those two sequence sets [23]. New algorithms to improve base calls from the raw data, as well as new experimental procedures are both under development that will better address this issue in future studies. HIV RT also introduces frameshifts and stop codons on its own, so it is unclear whether a given error was introduced naturally by HIV *in vivo* or through the sequencing protocol. Both are no doubt contributing to the observed outcome. We identified similar problem sequences in our control experiments that were based on mixtures of cloned DNA (described below).

What we were left with after filtering was an essentially intact set of V3 amino sequences, compressed, so that each unique form was represented once, and each sequence was named for the sample, the number of times it was found in the sample, and then ordered from most to least common. These sets were used for all subsequent analyses.

### Amplicon Resequencing Control Experiments

A series of control experiments that determined the accuracy, precision, and reproducibility of quantitative deep sequencing are described in **Supplementary Methods S1**, **sections 2 and 3**.

### Coreceptor Usage Prediction

Coreceptor usage for subjects 18, 19 and 47 was predicted using the position-specific scoring matrices (PSSM) [32]. For subject 07, coreceptor usage was determined phenotypically by testing recombinant chimeric viruses that incorporated subject-derived *env*s on CCR5- and CXCR4-expressing cell lines [21].

### Deep Sequence Data Sets

Raw sequence files from this study have been deposited in the NCBI Short Read Archive (http://www.ncbi.nlm.nih.gov/Traces/sra/sra.cgi?cmd=table&f=sample&m=data&s=sample), accession numbers SRS000811-000829.

## Supporting Information

Supplementary Methods S1 (0.22 MB DOC)

Figure S1 Maximum likelihood trees by PhyML of two control experiments. The comparison of shifts in the entropy of the subject samples were based on the full sequence set, and so we determined the entropy in each of the control experiments that was contributed by experimental error, not the input diversity. The entropy contributed by the experimental error in the two control experiments was calculated by combining the sequences that matched an input sequence as one category. The two controls were indistinguishable, and had significantly lower entropy than even the most conserved of the subject's samples: control experiment 1, H = 0.401 [95% CI, 0.377–0.413 (based on 100 bootstrap resamplings)]; control experiment 2: H = 0.414 [95% CI, 0.396–0.426].
(0.46 MB TIF)

Figure S2 Neighbor joining trees of all unique sequences in control experiments 1 and 2. The input sequences A, B and C are labeled in color and their locations are shown with vertical bars in the trees. A star denotes probable recombinants and # highlights two very divergent sequences in experiment 2.
(0.16 MB TIF)

Figure S3 Neighbor-joining (NJ) trees over time. NJ trees for (A) subject 07, (B) subject 19, and (C) subject 47 include all unique V3 forms found in each subject and also indicate the frequency of the 3 most common nucleotide forms at each time point. The most common sequence at the first time point was used as an out-group for the trees. For sub19 detail see Fig S5.
(0.73 MB TIF)

Figure S4 Maximum likelihood (ML) trees of the unique V3 forms. The ML trees include only sequences found more than 0.1% of the time for (A) subject 07, (B) subject 19, and (C) subject 47. Amino acid sequences at branch points are shown. Week 0, yellow; intermediate time point, red; VF, blue.
(0.43 MB TIF)

Figure S5 Neighbor joining tree detail, subject 19. Quantitative dynamic changes in V3 forms are highlighted through 17 weeks of VCV therapy. Rare forms can exhibit dramatic proportional increases or persist as minor forms; these forms acquire variants over time, suggesting active replication. Other rare forms can be lost entirely from the population. The absolute numbers of V3 loop forms are shown. The total numbers of V3 forms sequenced for each time point are shown in Table S1.
(0.84 MB TIF)

Table S1 (0.03 MB DOC)

Table S2 (0.03 MB DOC)

Table S3 (0.05 MB DOC)

Table S4 (0.04 MB DOC)

Table S5 (0.06 MB DOC)

Table S6 (0.04 MB DOC)
