# Supplementary material for: Quantitative Deep Sequencing Reveals Dynamic HIV-1 Escape and Large Population Shifts during CCR5 Antagonist Therapy In Vivo
Source: PLoS One. 2009 May 25;4(5):e5683. doi: 10.1371/journal.pone.0005683 (PMC2682648; doi:10.1371/journal.pone.0005683)
Supplement: Supplementary Methods S1 — (0.22 MB DOC) [file pone.0005683.s001.doc]

**Supplementary Information for:**

**Quantitative deep sequencing reveals dynamic HIV-1 escape and large population shifts during CCR5 antagonist therapy *in vivo***

Athe M.N. Tsibris^1,2^, Bette Korber^3,4†^, Ramy Arnaout^5,6,7†^, Carsten Russ^7^, Chien-Chi Lo^3^, Thomas Leitner^3^, Brian Gaschen^3^, James Theiler^3^, Roger Paredes^2,5,8^, Zhaohui Su^9^, Michael D. Hughes^9^, Roy M. Gulick^10^, Wayne Greaves^11^, Eoin Coakley^12^, Charles Flexner^13^, Chad Nusbaum^7^, Daniel R. Kuritzkes^2,5*^

*^1^Massachusetts General Hospital, Boston, MA, USA; ^2^Harvard Medical School, Boston, MA, USA;* *^3^Los Alamos National Laboratories, Los Alamos, NM, USA;  ^4^Santa Fe Institute, Santa Fe, NM, USA; ^5^Brigham and Women’s Hospital, Boston, MA, USA; ^6^Program for Evolutionary Dynamics, Harvard University, Cambridge, MA, USA; ^7^Broad Institute of* *MIT and Harvard, Cambridge, MA, USA; ^8^Fundacions irsiCaixa i Lluita contra la SIDA, Hospital Universitari Germans Trias i Pujol, Universitat Autònoma de Barcelona, Badalona, Catalonia, Spain; ^9^Harvard School of Public Health, Boston, MA, USA; ^10^Weill Medical College, Cornell University, New York, NY, USA; ^11^Schering-Plough Research Institute, Kennilworth, NJ, USA; ^12^Monogram Biosciences, South San Francisco, CA, USA; ^13^Johns Hopkins University, Baltimore, MD.*

^†^Both authors contributed equally to this work.

**TABLE OF CONTENTS**

**Page Number**

**Title 1**

**Table of Contents 2**

**Materials and Methods**

**1. Laboratory Techniques**

1.1. Sample Preparation **3**

1.2 Choice of Amplification Enzymes **3**

1.3 Real-time RT-PCR validation of initial viral template quantity **3**

**2. Control Experiments – Amplicon Resequencing**

2.1. Accuracy **4**

2.2. Precision **5**

**3. PCR, Recombination, and 454 Sequencing Bias**

3.1 Experimental Design **6**

3.2 Alignment and Filtering **7**

3.3 Consideration of Sequence Error and Impact on Analyses **8**

**References 11**

**Acknowledgements 11**

**Materials and Methods**

**1. LABORATORY TECHNIQUES**

**1.1. Sample Preparation.**

VViral RNA extraction Plasma volumes and corresponding HIV RNA copy numbers used for each sample prior to RNA extraction are shown below:


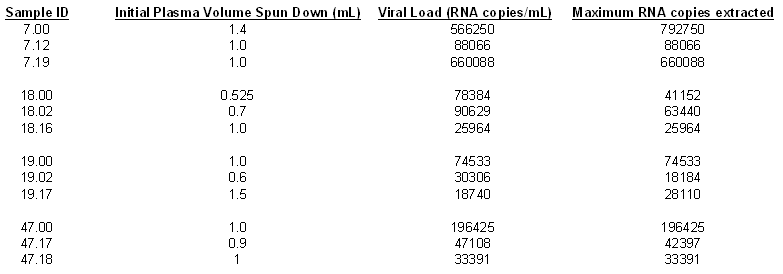


1.2 **Choice of amplification enzymes** The inherent error rate of the reverse transcriptase and polymerase used to synthesize and amplify cDNA can bias deep sequencing results. *Pfx* polymerase has a lower error rate than *Taq* polymerase, but no commercially available kit combines cDNA synthesis and *Pfx* polymerase amplification into a single step. Due to concerns about potential loss of DNA molecules with repeated extractions and purifications that would compromise our ability to detect minor variants, we elected to synthesize and amplify DNA in a single step, albeit using a polymerase with a slightly higher error rate. We then attempted to quantify this error in a series of deep sequencing control experiments.

1.3 **Real-time RT-PCR validation of initial viral template quantity.** The table below shows the volume of plasma used for RNA extraction and the expected RNA copy number in that volume based on results of commercial viral load testing. After accounting for the fractional volume of extracted RNA used in each real-time PCR reaction (10µL from a total of 40µL of eluted RNA), the table lists both the expected RNA copy number and the experimentally derived observation. Note that the expected RNA copy number represents the maximum RNA copy number possible and does not correct for either a loss of RNA with freeze-thaw or an extraction efficiency less than 100%.

| **Sample ID** | **Viral load (copies/mL)** | **Volume used for extraction (mL)** | **Maximum Expected Copy Number** | **RNA Fraction used for real-time RT-PCR** | **Expected Copy Number** | **Observed Copy Number** | **Template Proportion Amplified (%)** |
| --- | --- | --- | --- | --- | --- | --- | --- |
| 18.00 | 78,384 | 0.725 | 56,828 | 0.25 | 14,207 | 8,702 | 61.3 |
|  |  |  |  |  |  |  |  |
| 47.00 | 196,425 | 1.0 | 196,425 | 0.25 | 49,106 | 29,475 | 60.6 |
| 47.17 | 47,108 | 1.0 | 47,108 | 0.25 | 11,777 | 3,005 | 25.5 |

**2. CONTROL EXPERIMENTS – AMPLICON RESEQUENCING**

**2.1. Accuracy of *env* quasispecies quantification by deep sequencing.**

We first assessed the feasibility of using this novel sequencing technology to detect and quantify minority sequence variants in the V3 loop-coding segment of HIV-1 *env* with predicted tropism that differed from the majority populations. The V3 loop-coding region of HIV-1 env was amplified from 3 different viruses. The sources of the 3 amplicons were a laboratory-adapted virus known to use CCR5 exclusively (designated JC5) and 2 distinct clinical isolates, one an R5 virus (designated 34F) and the other an X4 virus (designated 352) as predicted by the position-specific scoring matrix (PSSM). Additionally, sample 34F was CCR5-using by the Trofile assay. The V3 loop sequences were amplified from full-length *env* clones using amplicon-specific primers. The three resulting amplicons were purified and pooled in defined proportions of 89%, 10% and 1%, respectively, and submitted to collaborators at the Broad Institute as a blinded mixture for analysis by ultradeep sequencing. The alignment below provides the nucleotide and amino acid sequences of the three V3 loop amplicons included in this pilot experiment.

**Nucleotide and amino acid sequence of the three amplicons. JC5, majority R5 sequence. 34F, minority R5 sequence. 352, minority X4 sequence.**


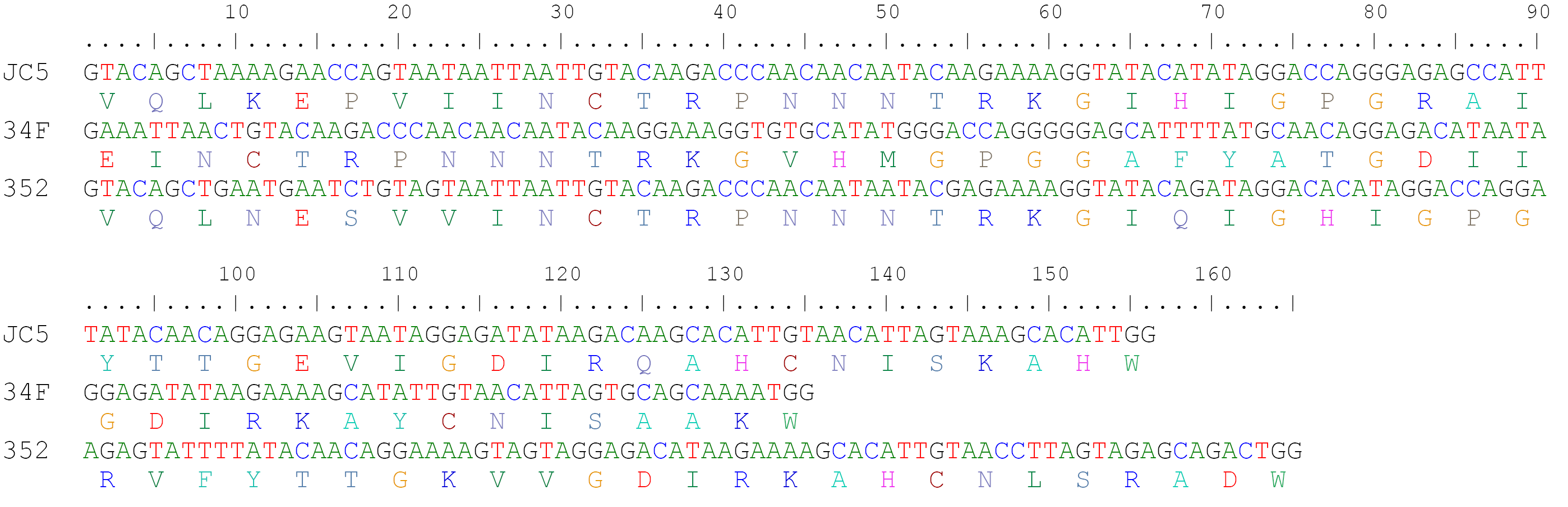


A total of 14,662 sequences were obtained from the pooled PCR products. Sequences were assigned using in-house code built around NCBI BLAST 2.2.10. The proportion of assigned sequences corresponding to the majority R5 sequence, the minority R5 sequence and the minority predicted X4 sequence were 89.6 ± 0.6% (mean ± SD), 9.3 ± 0.6% and 1.1 ± 0.1%, respectively. In our pilot experiment, deep sequencing accurately and quantitatively detected the minority V3 loop sequences from a mixture of amplicons.

**2.2 Precision of deep sequencing quantification.**

Source RNA for this experiment was a baseline (week 0) plasma sample of subject 18. Tropism testing (Trofile) detected only R5 virus at baseline, but a dominant CXCR4-using population (based on Trofile assay and a clonal V3 loop sequence analysis) emerged at a later time point. HIV-1 RNA was extracted from the week 0 plasma sample, reverse transcribed and amplified over 30 cycles of conventional PCR using patient-specific primers. After purification, the first round product served as template for an additional 30-cycle PCR reaction using patient-specific inner primers that generated 155-nucleotide amplicons containing the V3 loop. The V3 loop amplicons from 4 identical amplifications were submitted separately to the Broad Institute for 454 sequencing analysis, performed at 10,000x coverage per sample. We then used in-house code to assign sequences and determine the proportion of the later CXCR4-using sequence variants present in each replicate of the week 0 sample. Results are shown below:

Reproducibility of sequence proportions under identical conditions using 454 sequencing.

| Amplification Replicate | Number of Reads | Number of minor variant reads | % Minor variant reads |
| --- | --- | --- | --- |
| 1 | 9,669 | 222 | 2.30 |
| 2 | 13,160 | 424 | 3.22 |
| 3 | 8,541 | 169 | 1.98 |
| 4 | 11,036 | 240 | 2.17 |
| Mean (±S.D.) | 10,602±1867 | 264±111 | 2.42% ±0.55% |

The mean percentage (± SD) of the minor CXCR4-using variant was 2.42% ± 0.55%, and the coefficient of variation was 22.8%.

**3. PCR, recombination, and sequence error**

**3.1 Experimental Design.**

We initially considered using emulsion PCR to limit proportion bias and recombination frequency. To assess the need for emulsion PCR, we performed a pilot experiment to determine whether proportion bias was introduced into samples by conventional PCR. We pooled 3 plasmids containing defined proportions of clonal V3 loop sequences derived from subject 07 (SeqA, SeqB, and SeqC at 89%, 1% and 10%, respectively) and then performed 30 cycles of conventional PCR. Note that in this experiment, in contrast to the one described above, Sequences A, B, and C were pooled *prior* to PCR amplification. To eliminate length bias, the three V3 sequences used were each 174 nucleotides in length (see below). To eliminate primer bias, these sequences had common 5’- and 3’- ends, allowing us to use one set of primers to amplify all 3 sequences. Sequence A and B differed by 10 nucleotides, sequence A and C by 3 nucleotides, and sequences B and C by 9 nucleotides.

**Nucleotide sequences of the three V3 loop amplicons used in a conventional PCR pilot experiment**

The plasmid mixture containing the V3 sequences of interest was diluted to 10^4^ copies/PCR amplification (an input copy number similar to that of HIV-1 RNA in samples from A5211 subjects) and amplified by conventional PCR for 30 cycles. This experiment was repeated twice to assess reproducibility. The V3 loop amplicons were purified by gel extraction and the PCR product was submitted in blinded fashion for 454 sequence analysis to determine if the original sequence proportions had been maintained. Both sample replicates were sequenced to a depth of >50,000x coverage and sequences were assigned to one of the three input sequences using in-house code.

**3.2. Alignment and filtering.**

We applied the same filtering and alignment strategy that we developed for the subject samples to the controls:

| **Subject Week** | **Reads** | **Cut: < 70% similarity to HXB2 (uniq/reads)** | **Cut: <123 nt (uniq/reads)** | **Aligned DNA Sequence number (uniq/reads)** | **Frameshifts Stop Codons Missing sequence** | **Final** | **Uniq** |
| --- | --- | --- | --- | --- | --- | --- | --- |
| Control 1 | 53,898 | 70 | 1,355 | 52,473 | 3684 | 48,789 | 307 |
| Control 2 | 57,257 | 105 | 1,522 | 55,630 | 6802 | 48,828 | 322 |

The data obtained with the final V3 loop sequences listed above are shown in Table S4. Out of the sequences that were retained through our filtering process, over 95% of the sequences perfectly matched one of the input strains in the target V3 region at the amino acid level, which was the most relevant measure, as that is what we used for our analysis (this approach does not account for silent mutations, however). The retention of the input ratio 89:10:1 was well preserved, indicating our filtering process did not introduce significant bias; only ~4.5% of the sequences in the control experiments were mismatches relative to the input.

**3.3. Considerations of sequence error and impact on analyses**

Both biological error introduced by HIV-1 RT and experimental error introduced at the amplification and 454 sequencing stages contributed to the apparent diversity in our subject samples, and it is difficult to differentiate between these sources of error. The control experiments, however, indicate that after the pre-filtering step we took to exclude overtly damaged sequences, over 95% of the observed control sequences accurately reflected the input data, and importantly, the filtering did not bias the relative frequencies of the sequences. Thus, most of the sequences are valid, or have minimal error. After filtering out clearly problematic sequences, the per base error was low, between 0.0011 and 0.0016.

We have considered the subject-derived plasma data in two ways. First, we looked at the intact data set. While some of the very rare and unusual sequences were the consequence of experimental error, other rare sequences were interesting in terms of the longitudinal data, and merited consideration. For example, the second most common sequence in the week 2 sample from subject 18 was found 1101 times, but was found only once at week 0. Similarly, the second most common sequence in the week 0 sample from subject 07 was found 35,676 times, but by week 19 was found only once. If we had excluded rare variants, we would not have detected these shifts in frequencies.

We also examined the data in ways that should exclude sequences with errors by reviewing changes in frequency of common forms. First we assessed the frequency changes in the 10-12 most common sequences found at each time point for each subject (**Fig. 2**). By virtue of frequent repetition in at least one of each subject’s samples, these sequences are all most likely viable, and the extreme shift in the frequencies of these sequences observed over time provided dramatic evidence of selection. We also used PhyML to produce maximum likelihood trees to trace evolutionary trajectory restricted to sequences present in over 0.1% of the sample, so that each individual sequence was likely to be a reflection of viable V3 sequences in the sample, and we tracked shifts in the magnitude of the frequency of the sequence (**Fig. 4 and S3**) ^1^. Despite this tree being a skeletal view of the full richness of diversity seen in the NJ tree that includes all sequences, this minimal tree still captured many of the major features of the evolutionary trajectory of the V3 loop in these patients. A comparable figure of the data from the two control samples (**Fig. S1**) revealed, as expected, a far simpler picture, with the three input sequences present in the appropriate ratios. Unique sequences that constituted more than 0.1% of the total population of sequences but contained an error are indicated; all common sequences with errors were just one amino acid different from the most common input strain. This control sequence tree is a simple visualization of the alignment and is revealing. It illustrates a much less complex sample than the subject samples, with neither accumulation of mutations nor development of alternate lineages evident. These trees were constructed using the default setting on PhyML for protein sequences ^2^.

All unique sequences from control experiment 1 and 2 were used to calculate separate neighbor-joining trees in the same way as the A5211 sequence data were investigated (**Fig S2**). The MASE amino acid alignments were converted to NEXUS format, and a neighbor joining tree based on total character differences was inferred using PAUP* ^3^. Since there were mostly single amino acid changes from the input sequences (A, B and C), we calculated neighbor joining trees with several different settings (zero branch length handling, gap treatment, tiebreaking, total or mean character differences); all gave trees with very similar relative branch lengths and topology (0-46 symmetric tree-to-tree differences). As can be seen in Figure S2, the three input sequences displayed some variants around them, and these were point mutation errors. There were also variants classified between the input sequences; these were likely amplification strand switches (recombinants). In addition, in control experiment 2, two very divergent sequences appeared, but it was unclear how they were formed. Encouragingly, the number of errors around each input sequence reflected the proportions by which they were mixed and the error ratios were nearly identical. After filtering and aligning the data from the first control experiment, the input sequences A:B:C input ratio 89:1:10 were well preserved at 88.5 : 0.93% : 10.4% with 0.15 being a clear recombinant between the most distinctive input sequence B and either A or C. In the second control experiment the ratio was: 86.7 : 1.7 : 11.7 with 0.11 being clear recombinants B and A or C recombinants.

**References**

1. Guindon, S. & Gascuel, O. A simple, fast, and accurate algorithm to estimate large phylogenies by maximum likelihood. *Systematic biology* **52**, 696-704 (2003).

2. Guindon, S., Lethiec, F., Duroux, P. & Gascuel, O. PHYML Online--a web server for fast maximum likelihood-based phylogenetic inference. *Nucleic acids research* **33**, W557-559 (2005).

3. Swofford, D.L. PAUP* Phylogenetic Analysis Using Parsimony (*and Other Methods). (Sinauer Associates, Sunderland, MA, 2002).

GeneCutter: Sequence Alignment and Protein extraction

(http://H[www.hiv.lanl.gov/content/sequence/GENE_CUTTER/cutter.html](http://www.hiv.lanl.gov/content/sequence/GENE_CUTTER/cutter.html)H)

**Acknowledgements**

We thank the participating subjects, the AIDS Clinical Trials Group protocol A5211 team, and the participating sites for their efforts during this clinical trial. We thank Schering-Plough for providing vicriviroc. We thank Charles Calef for help with programming. Funding support: AMNT (Clinical Investigator Training Program: Harvard/MIT Health Sciences and Technology-Beth Israel Deaconess Medical Center, in collaboration with Pfizer Inc. and Merck and Co.), and P30 AI060354); DRK (R37 AI553537, K24 RR016482, and U01 AI069472); R.M.G. (K24 AI51966, U01 AI069419); the AIDS Clinical Trial Group (U01 AI068636). RP is a recipient of “*La Caixa*” Fellowship Grant for Post-Graduate Studies, *Caixa d’Estalvis i Pensions de Barcelona, “La Caixa”*, Barcelona, Catalonia, Spain. Analysis was supported by Center for HIV/AIDS Vaccine Immunology (U01 AI067854, BK, BG), and through LANL LDRD research and development support (BK, CC, TL).
