# Supplementary figures and images for: Quantitative Deep Sequencing Reveals Dynamic HIV-1 Escape and Large Population Shifts during CCR5 Antagonist Therapy In Vivo
Source: PLoS One. 2009 May 25;4(5):e5683. doi: 10.1371/journal.pone.0005683 (PMC2682648; doi:10.1371/journal.pone.0005683)

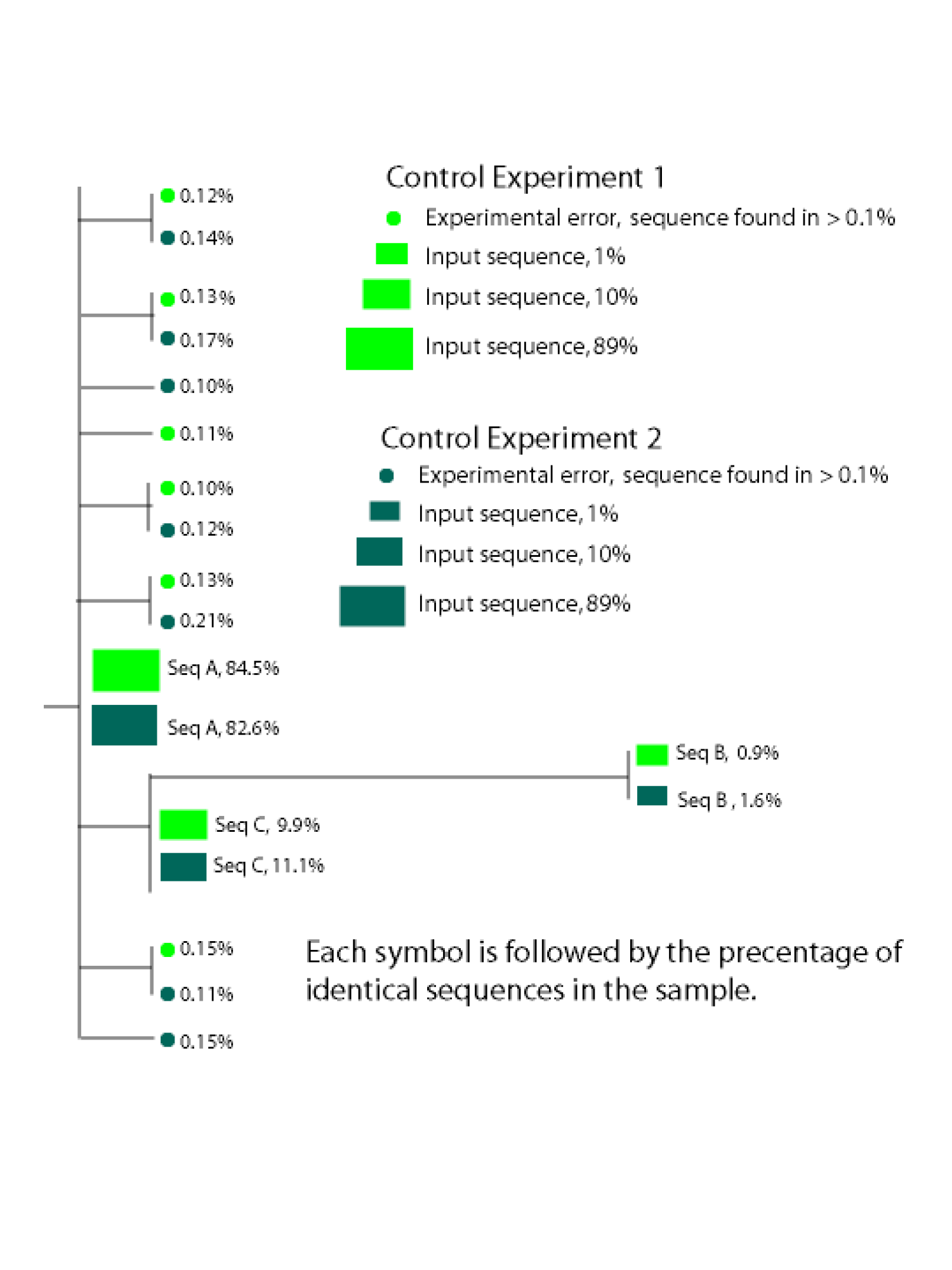

Supplement: Figure S1 — Maximum likelihood trees by PhyML of two control experiments. The comparison of shifts in the entropy of the subject samples were based on the full sequence set, and so we determined the entropy in each of the control experiments that was contributed by experimental error, not the input diversity. The entropy contributed by the experimental error in the two control experiments was calculated by combining the sequences that matched an input sequence as one category. The two controls were indistinguishable, and had significantly lower entropy than even the most conserved of the subject's samples: control experiment 1, H = 0.401 [95% CI, 0.377–0.413 (based on 100 bootstrap resamplings)]; control experiment 2: H = 0.414 [95% CI, 0.396–0.426]. (0.46 MB TIF) [file pone.0005683.s002.tif]

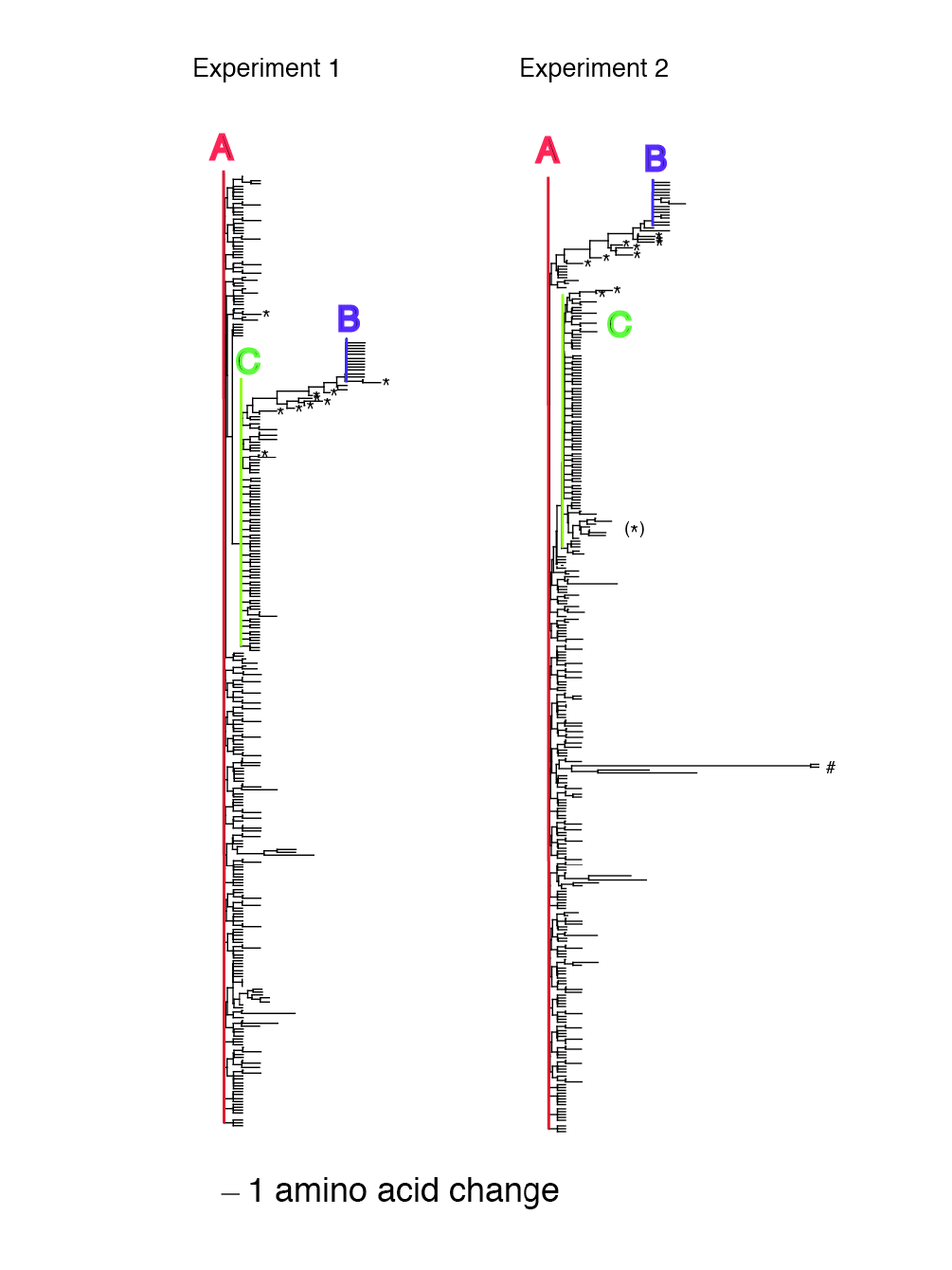

Supplement: Figure S2 — Neighbor joining trees of all unique sequences in control experiments 1 and 2. The input sequences A, B and C are labeled in color and their locations are shown with vertical bars in the trees. A star denotes probable recombinants and # highlights two very divergent sequences in experiment 2. (0.16 MB TIF) [file pone.0005683.s003.tif]

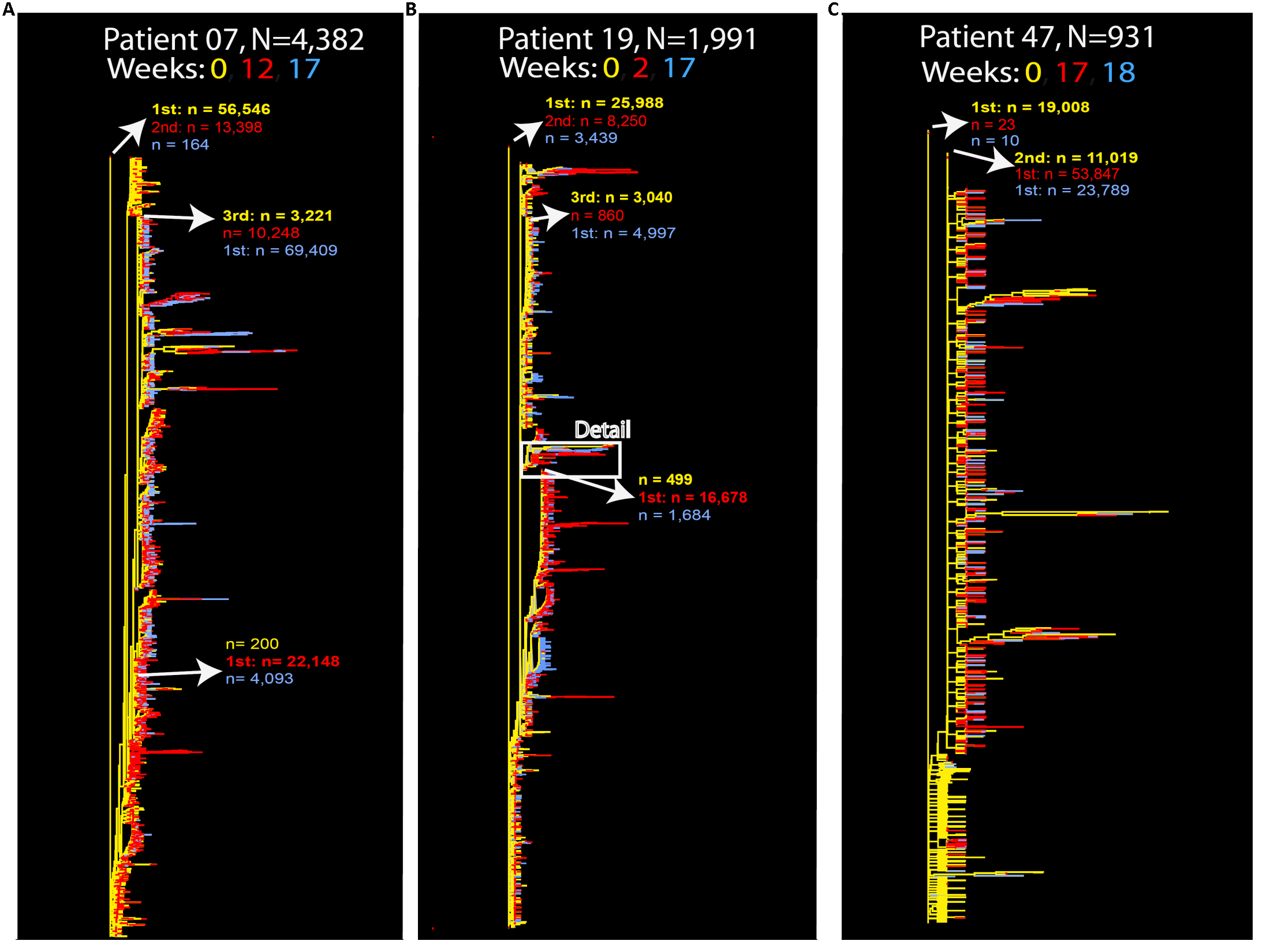

Supplement: Figure S3 — Neighbor-joining (NJ) trees over time. NJ trees for (A) subject 07, (B) subject 19, and (C) subject 47 include all unique V3 forms found in each subject and also indicate the frequency of the 3 most common nucleotide forms at each time point. The most common sequence at the first time point was used as an out-group for the trees. For sub19 detail see Fig S5. (0.73 MB TIF) [file pone.0005683.s004.tif]

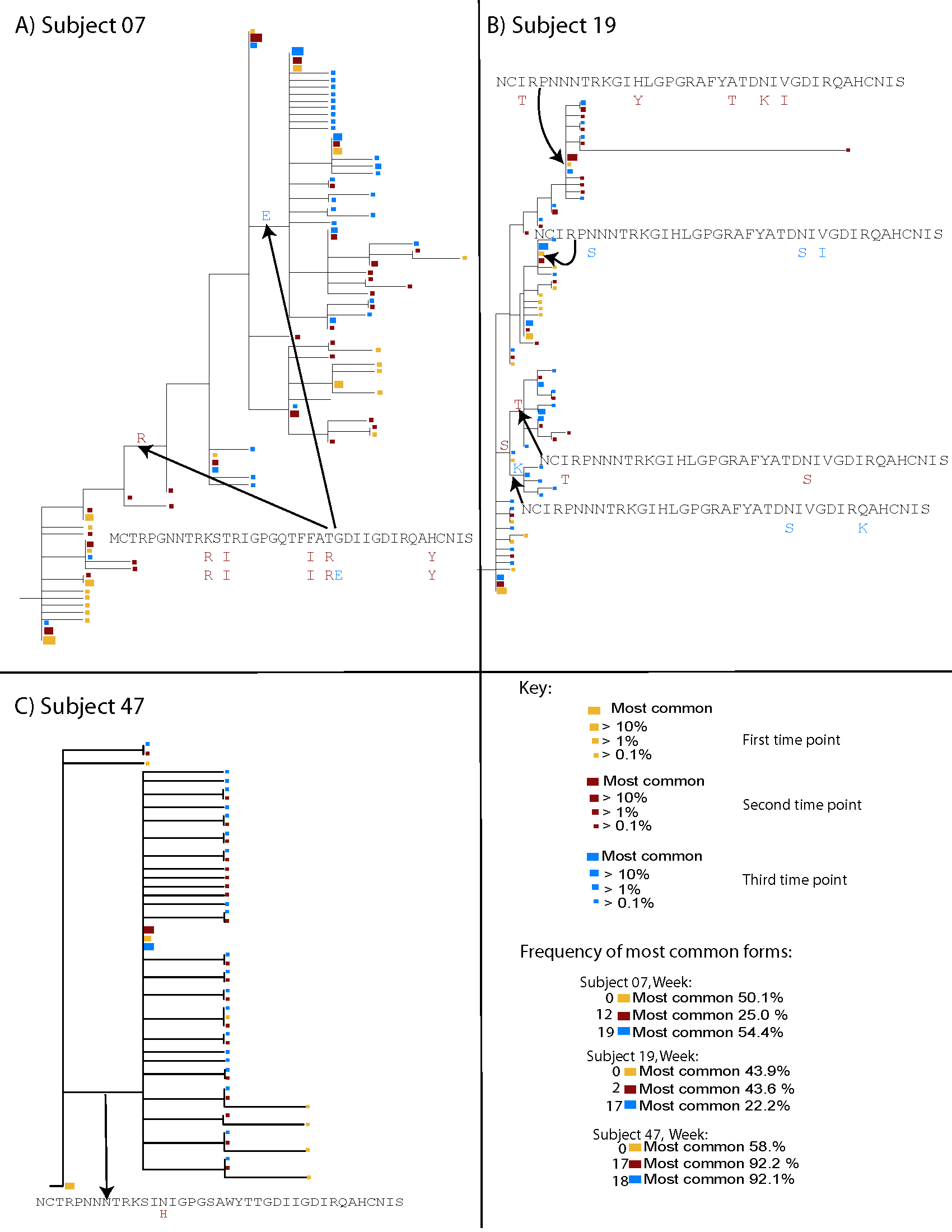

Supplement: Figure S4 — Maximum likelihood (ML) trees of the unique V3 forms. The ML trees include only sequences found more than 0.1% of the time for (A) subject 07, (B) subject 19, and (C) subject 47. Amino acid sequences at branch points are shown. Week 0, yellow; intermediate time point, red; VF, blue. (0.43 MB TIF) [file pone.0005683.s005.tif]

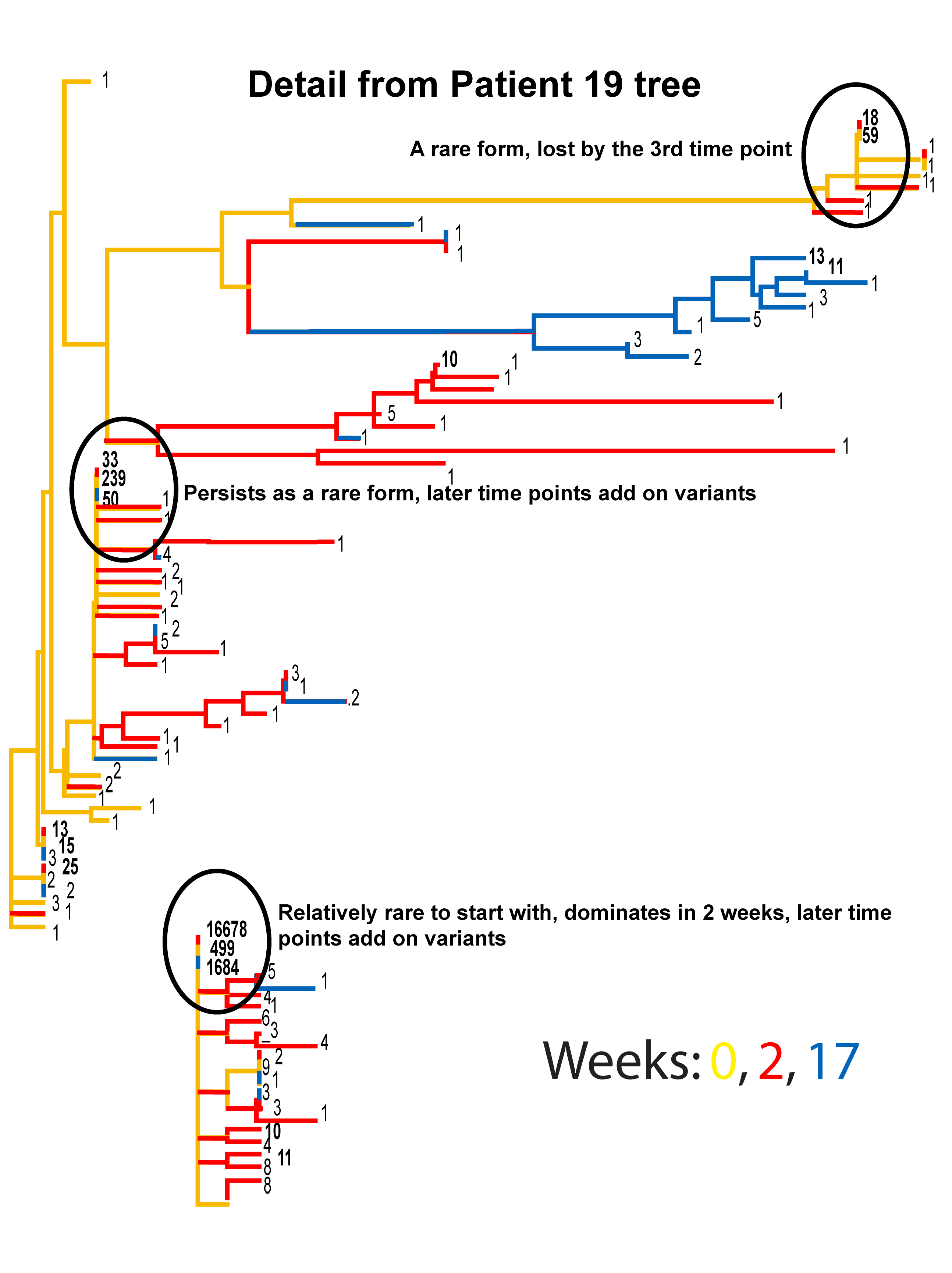

Supplement: Figure S5 — Neighbor joining tree detail, subject 19. Quantitative dynamic changes in V3 forms are highlighted through 17 weeks of VCV therapy. Rare forms can exhibit dramatic proportional increases or persist as minor forms; these forms acquire variants over time, suggesting active replication. Other rare forms can be lost entirely from the population. The absolute numbers of V3 loop forms are shown. The total numbers of V3 forms sequenced for each time point are shown in Table S1. (0.84 MB TIF) [file pone.0005683.s006.tif]
