# Supplementary material for: Quantitative Deep Sequencing Reveals Dynamic HIV-1 Escape and Large Population Shifts during CCR5 Antagonist Therapy In Vivo
Source: PLoS One. 2009 May 25;4(5):e5683. doi: 10.1371/journal.pone.0005683 (PMC2682648; doi:10.1371/journal.pone.0005683)
Supplement: Table S1 — (0.03 MB DOC) [file pone.0005683.s007.doc]

**Table S1. Control Experiments – Effect of amplification and 454 Sequencing on Amplicon Quantification and Error Rates**

Experiment Number of perfect matches, % of perfect matches Total, perfect matches Mismatches

­­­­­­­_________ _______________________________________________ __________________ ____________

A B C

Control 1 41,316 (88.7%) 432 (0.93%) 4,852 (10.4%) 46,600 (95.5%) 2,189 (4.5%)

Control 2 40.330 (86.6%) 790 (1.7%) 5,456 (11.7%) 46,576 (95.4%) 2,252 (4.6%)

The mismatched sequences were for the most part only one amino acid different than one of the three input strains; only 0.1% and 0.2% of the mismatched sequences differed by more than one amino acid from one of the input sequences. If each of the sequences with a sequencing error was grouped with the input sequence to which it was most similar, the 89:10:1 ratio was very well preserved. A small number of the sequences were deemed most likely to be in vitro recombinants between sequence B and sequence A or C (sequences A and C were too similar to detect recombinants). These designations were given when a single or double recombination breakpoint could explain the observed sequence as well as base substitution.

Experiment Number matching or similar, % of total Recombinant Total

­­­­­­­_________ _______________________________________________ ___________ _______

A-like B-like C-like

Control 1 43182 (88.5%) 453 (0.93%) 5081 (10.4%) 73 (0.15%) 48,789

Control 2 42218 (86.4%) 834 (1.7%) 5724 (11.7%) 52 (0.11%) 48,828
