# Supplementary material for: Quantitative Deep Sequencing Reveals Dynamic HIV-1 Escape and Large Population Shifts during CCR5 Antagonist Therapy In Vivo
Source: PLoS One. 2009 May 25;4(5):e5683. doi: 10.1371/journal.pone.0005683 (PMC2682648; doi:10.1371/journal.pone.0005683)
Supplement: Table S2 — (0.03 MB DOC) [file pone.0005683.s008.doc]

**Table S2**. **Proportion retention and sequence error in control experiments with 454 sequencing**

A) Input strains:

SeqA.Exp1 41316 84.7% MCTRPGNNTRKSTRIGPGQTFFATGDIIGDIRQAHCNIS

SeqA.Exp2 40330 82.6% ---------------------------------------

SeqB.Exp1 432 0.9% ----------RPI--------I-RE---------Y----

SeqB.Exp2 790 1.6% ----------RPI--------I-RE---------Y----

SeqC.Exp1 4852 9.9% ----------R----------------------------

SeqC.Exp2 5456 11.2% ----------R----------------------------

Most common errors:

A-like.Exp2 60 0.12% L--------------------------------------

A-like.Exp1 70 0.14% L--------------------------------------

A-like.Exp1 66 0.13% -----------G---------------------------

A-like.Exp2 103 0.21% -----------G---------------------------

A-like.Exp2 73 0.15% -------------G-------------------------

A-like.Exp2 49 0.10% ---------------------L-----------------

A-like.Exp2 63 0.13% -------------------------G-------------

A-like.Exp1 83 0.17% -------------------------G-------------

A-like.Exp1 51 0.10% --------------------------V------------

A-like.Exp2 57 0.12% --------------------------V------------

A-like.Exp1 75 0.15% -----------------------------G---------

A-like.Exp2 54 0.11% -----------------------------G---------

A-like.Exp1 55 0.11% -------------------------------G-------

B) Common errors at the codon level

1. ATG (M) -> CTG (L) A->C (both sets)

2. AGT (S) -> GGT (G) A->G (both sets)

3. AGA (R) -> GGA (G) A->G (exp 2)

4. TTT (F) -> CTT/TCT(L) T->C (exp 2)

5. GAC (D) -> GGC (G) A->G (both sets)

6. ATA (I) -> GTA (V) A->G (both sets)

7. GAC (D) -> GGC (G) A->G (both sets)

8. AGA (R) -> GGA (G) A->G (exp 1)

A) An alignment of the sequences that were present in > 0.1% of the sequences from the two control experiments, starting with the three input strains, and the number of times each was observed, for those sequences that were present in > 0.1% of the filtered final alignment. 13 substitutions were found to recur more than 0.1% of the time in one or the other sets, and 5 of these appeared in both sets, suggesting a predilection for error at that site, B) Most common errors at the codon level; most were A🡪G mutations. The codons represent the most common errors, ordered from left to right across the V3 loop.
