# Supplementary material for: Quantitative Deep Sequencing Reveals Dynamic HIV-1 Escape and Large Population Shifts during CCR5 Antagonist Therapy In Vivo
Source: PLoS One. 2009 May 25;4(5):e5683. doi: 10.1371/journal.pone.0005683 (PMC2682648; doi:10.1371/journal.pone.0005683)
Supplement: Table S3 — (0.05 MB DOC) [file pone.0005683.s009.doc]

Table S3. Sequential filtering of raw 454-derived V3 loop nucleotide sequences.

| **Subject Week** | **Reads** | **Cut:**  **< 70% similarity to HXB2 (reads)** | **Cut: <123 nt (reads)** | **Aligned DNA Sequence number (reads)** | **Cut Frame shifts, Stop Codons Large gaps** | **Final** | **Unique** |
| --- | --- | --- | --- | --- | --- | --- | --- |
| 0700 | 125,299 | 112 | 4,144 | 121,043 | 8,225 | 112,818 | 1,151 |
| 0712 | 120,539 | 107 | 4,657 | 115,775 | 27,091 | 88,684 | 1,818 |
| 0719 | 135,649 | 109 | 3,900 | 131,640 | 4144 | 127,496 | 1,413 |
| 1800 | 138,681 | 1,275 | 7,138 | 130,268 | 19797 | 110,471 | 1,101 |
| 1802 | 62,475 | 608 | 9,464 | 52,403 | 26984 | 25,419 | 363 |
| 1816 | 98,025 | 1,074 | 10,599 | 86,392 | 72026 | 14,366 | 446 |
| 1816** |  |  |  |  |  | 48,862 | 726 |
| 1900 | 70,391 | 1,450 | 3,963 | 64,978 | 5752 | 59,226 | 613 |
| 1902 | 46,826 | 360 | 3,242 | 43,224 | 5007 | 38,217 | 719 |
| 1917 | 25,685 | 449 | 1,481 | 23,755 | 1236 | 22,519 | 659 |
| 4700 | 36,889 | 8 | 2,025 | 34,856 | 2653 | 32,203 | 346 |
| 4717 | 64,253 | 6 | 1,952 | 62,295 | 3913 | 58,382 | 350 |
| 4718 | 28,445 | 11 | 771 | 27,663 | 1837 | 25,826 | 235 |

This table lists how many sequences were removed by each criteria and the number of sequences remaining after each of the filtering steps. We cut the number of sequences by the red value for every case – the first two steps were at the nucleotide level, the last cut after the protein translation, and the resulting amino acid sequence alignment were used for subsequent analyses. ** In sample 1816, 48,862 sequences were excluded, mostly as a result of a single base deletion frameshift in a polyA stretch. Many sequences were also lost in the other two samples from subject 18 for the same reason.
