# Supplementary material for: Quantitative Deep Sequencing Reveals Dynamic HIV-1 Escape and Large Population Shifts during CCR5 Antagonist Therapy In Vivo
Source: PLoS One. 2009 May 25;4(5):e5683. doi: 10.1371/journal.pone.0005683 (PMC2682648; doi:10.1371/journal.pone.0005683)
Supplement: Table S4 — (0.04 MB DOC) [file pone.0005683.s010.doc]

**Table S4. Longitudinal changes in baseline predicted CXCR4-using V3 forms: Sub18**

**Week 0 V3 Sequence Week 2 Week 16**

1800.0007_925 CERPNNNTRQRLSIGPGRSFYTSRRIIGDVKKAHC 1802.0001_21513 1816.0001_9811

1800.0057_45 CMRPNNNTRKSISIGPGRAFYTTGKIIGDIRQAHC none none

1800.0077_34 CIRPNNNTRQRLSIGPGRSFYTSRRIIGDVKKAHC 1802.0016_41 1816.0019_44

1800.0161_15 CIRPNNNTRKSISIGPGRSFYTSRRIIGDVKKAHC none none

1800.0165_15 CIRPNNNTRKSISIGPGRAFYTSRRIIGDVKKAHC 1802.0241_1 1800.0165_15

1800.0176_13 CERPNNNTRQRLSIGPGRSFYTSRRIIGDIRQAHC 1802.0169_2 none

1800.0223_9 CMRPNNNTRQRLSIGPGRSFYTSRRIIGDVKKAHC 1802.0035_24 none

1800.0234_8 CMRPNNNTRKSISIGPGRSFYTSRRIIGDVKKAHC none none

1800.0254_7 CERPNNNTRQRLSIGPGRAFYTTGEIIGDIRQAHC 1802.0076_8 none

1800.0262_6 CERPNNNTRQRLSIGPGRSFYTSRRIIGDVKRAHC 1802.0031_27 1816.0018_48

1800.0267_6 CERPNNNTRQRLSIGPGRSFYTSRRIVGDVKKAHC 1802.0006_110 1816.0021_38

1800.0281_6 CIRPNNNNKKKYIYRDQGESFYTTGEIIGDIRQAHC none none

1800.0296_5 CMRPNNNTRKSISIGPGRAFYTSRRIIGDVKKAHC none none

1800.0300_5 CERPNNNTRQRLSIGPGRSLYTSRRIIGDVKKAHC 1802.0023_32 1816.0034_18

1800.0342_3 CERPNNNTRQRLSIGPGRSFYTTGEIIGDIRQAHC 1802.0074_8 none

1800.0343_3 CERPNNNTRQRISIGPGRAFYTTGEIIGDIRQAHC 1802.0358_1 none

1800.0346_3 CVRPNNNTRQRLSIGPGRSFYTSRRIIGDVKKAHC 1802.0014_42 1816.0391_1

1800.0359_3 CERPNNDTRQRLSIGPGRSFYTSRRIIGDVKKAHC 1802.0019_40 1816.0063_7

1800.0361_3 CERPNNSTRQRLSIGPGRSFYTSRRIIGDVKKAHC 1802.0040_22 1816.0037_13

1800.0393_2 CNNEPNKQYKKSISIGPGRAFYTTGEIIGDIRQAHC none none

1800.0395_2 CERPSNNTRQRLSIGPGRSFYTSRRIIGDVKKAHC 1802.0025_32 1816.0045_11

1800.0397_2 CIRPNNSTRQRLSIGPGRSFYTSRRIIGDVKKAHC none none

1800.0435_2 CERPDNNTRQRLSIGPGRSFYTSRRIIGDVKKAHC 1802.0026_30 1816.0106_4

1800.0448_2 CMRPNNNTRKSIAIGPGRAFYTTGKIIGDIRQAHC none none

1800.0461_2 CMRPNNNNKKKYIYRDQGESFYTTGEIIGDIRQAHC none none

1800.0471_2 CERPNNNTRQRLSIGPGRSFYTSRRIIGDVKKAHC none none

1800.0480_2 RERPNNNTRQRLSIGPGRSFYTSRRIIGDVKKAHC 1802.0024_32 1816.0041_11

1800.0519_2 CERPNNNTRQRLSIGPGRSFYTSRRITGDVKKAHC 1802.0068_10 1816.0069_6

1800.0534_1 CERPNNNTRQRISIGPGRAFYATGEIIGDIRQAHC none none

1800.0539_1 CNNEPKQTITKKSISIGPGRAFYTTGEIIGDIRQAHC none none

1800.0551_1 CMRPNNNTRKSISIGPGRAFLYNRRIIGDIRQAHC none none

1800.0567_1 CIRPNNNTRRSISIGPGRAFYTTGKIIGDIRQAHC none none

1800.0572_1 CERPNNNTRKSISIGPGRAFYTSRRIIGDVKKAHC none none

1800.0578_1 CERPNNNTRQRPSIGPGRSFYTSRRIIGDVKKAHC 1802.0036_24 1816.0073_6

1800.0581_1 CMRPSNNTRKSISIGPGRAFYTTGKIIGDIRQAHC none none

1800.0587_1 CIRPTNNTRKSISIGPGRAFYTSRRIIGDVKKAHC none none

1800.0592_1 CERPNNNTRQRLSIGSGRSFYTSRRIIGDVKKAHC 1802.0131_3 1816.0368_1

1800.0593_1 CERPNNNTRQRLSIGPGRLFYTSRRIIGDVKKAHC 1802.0128_3 1816.0032_18

1800.0597_1 CMTTQQTITKKSISIGPGRAFYTTGDIIGDIRQAHC none none

1800.0618_1 CMRPNNNTRKSIPIGPGRAFYTSRRIIGDVKKAHC none none

1800.0633_1 CMRPNNNTRKSISIGPGRAFYTTGKIIGNIRQAHC none none

1800.0667_1 CIRPNNNTRKSISIGPGRAFLYNRRIIGDIRQAHC none none

1800.0686_1 CERPNNNTRQRLSIGPGRSFYTSGRIIGDVKKAHC 1802.0043_19 1816.0030_18

1800.0692_1 CERPNNNTRQRLSIGPGRSFYTSRRIIGDVKKAHC 1802.0107_4 none

1800.0699_1 CIRPNDNTRQRLSIGPGRSFYTSRRIIGDVKKAHC none none

1800.0719_1 CMRPNNNTRKSNIYRTRRAFYTTGDIIGDIRQAHC none none

1800.0732_1 CERPNDNTRQRLSIGPGRSFYTSRRIIGDVKKAHC 1802.0046_19 1816.0047_10

1800.0746_1 CERPNNNTRQRLSIEPGRSFYTSRRIIGDVKKAHC 1802.0081_7 none

1800.0754_1 CMRPNNNTRKSISIGPGRALYTSRRIIGDVKKAHC none none

1800.0792_1 RMRPNNNTRKSISIGPGRSFYTSRRIIGDVKKAHC none none

1800.0803_1 CERPNNNTRQRLSIGPGRSFYTSRRIIGDCKKAHY none none

1800.0806_1 CMRPNNNTRKSISIGPGRAFYTTRRIIGDVKKAHC none none

1800.0808_1 CERPNNNIRQRLSIGPGRSFYTSRRIIGDVKKAHC 1802.0070_10 1816.0049_9

1800.0825_1 CERPNNNTRQRLSTGPGRSFYTSRRIIGDVKKAHC 1802.0080_7 1816.0095_4

1800.0832_1 CNNEPKQTIQRKSISIGPGRAFYTTGEIIGDIRQAHC 1802.0346_1 none

1800.0845_1 CERPNNNTRQRLSIGPGRSFYTSRRIIGDVKQAHC none none

1800.0847_1 CIRPNNNTRQRLSMGPGRSFYTSRRIIGDIRQAHC none none

1800.0861_1 CERPNNNTRQRLSIGPGRSFYASRRIIGDVKKAHC 1802.0098_5 1816.0036_15

1800.0866_1 CIRPNNNTRKSISIGPGRSFYTSRRIIGDAKKAHC none none

1800.0869_1 CERPNNNTRQRLSIGPGRSFYTSRRIIGDSKKAHC 1802.0010_59 1816.0008_99

1800.0891_1 CMRPNNNTRKSISIGPERAFYTTGKIIGDIRQAHC none none

1800.0912_1 CERPNNNTRQRLSIGPGRPFYTPRRIIGDVKKAHC none none

1800.0915_1 CMRPNNNTRKSISIGPGRAFYTTGDIIGDIKTSTSC none none

1800.0926_1 CMRPNNNTRKSISIGPGRAFYTTGDIIGDIKTSTL none none

1800.0927_1 CERPNNNTRQRLSIGPGKSFYTSRRIIGDVKKAHC 1802.0045_19 1816.0033_18

1800.0955_1 CERPNNNTRQRLSIGPGRSFYTSRRIMGDVKKAHC 1802.0020_36 1816.0050_9

1800.0960_1 CERPNNNTRQRLSIGPGRSFYTSRRIIGDVKKAHC 1802.0057_13 1816.0070_6

1800.0968_1 CMRPNNNTRKSISMGPGRAFYTTGKIIGDIRQAHC none none

1800.0970_1 SERPNNNTRQRLSIGPGRSFYTSRRIIGDVKKAHC 1802.0078_7 none

1800.0975_1 CGRPNNNTRQRLSIGPGRSFYTSRRIVGDVKKAHC none none

1800.0992_1 CERPNNNTRRRLSIGPGRSFYTSRRIIGDVKKAHC 1802.0033_26 1816.0062_7

1800.1010_1 CGRPNNNTRQRLSIGPGRSFYTSRRIIGDVKKAHC 1802.0017_40 1816.0026_24

1800.1045_1 CERPNNNTRQRLSIGPGGSFYTSRRIIGDVKKAHC 1802.0077_7 1816.0011_83

1800.1050_1 CERPNNNTRQRLSIGPGRSFYTSRRIIGDVKKTHC 1802.0002_1101 1816.0005_267

1800.1051_1 CERPNNNTRQRLSIGPGRAFYTTGEIMGDIRQAHC none none

1800.1058_1 CMRPNNNTRKSISIGPGRAFYTTGKIIGDVRQAHC none none

1800.1068_1 CMRPNNNTRRSLSIGPGRSFYTSRRIIGDVKKAHC none none

1800.1073_1 CERPNNNTRQRLSIGPGSAFYTTGEIIGDIRQAHC none none

1800.1080_1 CMRPNNNTRKGISIGPGRAFYTTGKIIGDIRQAHC none none

1800.1086_1 CMRPNNNTRKSNIYRTRRAFYTTGEIIGDIRQAHC none none

Sequence names were written in the following way, 1900.0001_25988, means subject 19, week 00, .0001 means it was the most common sequence in the set, and 25988 is the number of times it was identically repeated in the 1900 sample.
