# Supplementary material for: Quantitative Deep Sequencing Reveals Dynamic HIV-1 Escape and Large Population Shifts during CCR5 Antagonist Therapy In Vivo
Source: PLoS One. 2009 May 25;4(5):e5683. doi: 10.1371/journal.pone.0005683 (PMC2682648; doi:10.1371/journal.pone.0005683)
Supplement: Table S5 — (0.06 MB DOC) [file pone.0005683.s011.doc]

Table S5. Longitudinal changes in baseline predicted CXCR4-using V3 forms: Sub19

| **Week 0** | **V3 Sequence** | **Week 2** | **Week 17** |
| --- | --- | --- | --- |
| 1900.0005_499 | CTRPNNNTRKGIYLGPGRAFYTTDKIIGDIRQAHC | 1902.0001_16678 | 1917.0005_1684 |
| 1900.0030_43 | CIRPNNNTRKGIYLGPGRAFYTTDKIIGDIRQAHC | 1902.0006_539 | 1917.0016_64 |
| 1900.0084_18 | CERPNNNTRQRLSIGPGRSFYTSRRIIGDVKKAHC | 1902.0016_59 | none |
| 1900.0207_5 | CIRPSNNTRKGIYLGPGRAFYTTDKIIGDIRQAHC | 1902.0076_14 | 1917.0034_20 |
| 1900.0225_4 | CTRPNNNTRRGIYLGPGRAFYTTDKIIGDIRQAHC | 1902.0004_1135 | 1917.0007_403 |
| 1900.0240_3 | CTRPNNNTRKGIYLGPGRAVYTTDKIIGDIRQAHC | None | none |
| 1900.0250_3 | CTRPNNNTGKGIYLGPGRAFYTTDKIIGDIRQAHC | 1902.0048_22 | 1917.0415_1 |
| 1900.0269_2 | CTRPNNNTRKGIYLGPGRAFYTTDKIIGDIRQAHC | 1902.0087_13 | none |
| 1900.0271_2 | CTRPNNNTRKGIYLGPGRAFYTTDKIIEDIRQAHC | 1902.0121_9 | 1917.0482_1 |
| 1900.0328_2 | CTRPNNNTRKGIYLGPGRAFYTTDKIIGDIRQAHW | 1902.0018_54 | none |
| 1900.0330_2 | CTRPNNNTRKGIYLGPGRAFYTTDKIIGGIRQAHC | 1902.0021_49 | 1917.0175_4 |
| 1900.0378_1 | CTRPNNSTRKGIYLGPGRAFYTTDKIIGDIRQAHC | 1902.0059_20 | 1917.0419_1 |
| 1900.0393_1 | CTRPNNNTRKGTYLGPGRAFYTTDKIIGDIRQAHC | 1902.0061_18 | 1917.0526_1 |
| 1900.0410_1 | CERPNNNTRQRLSIGPGRSFYTSRRIIGDVKKTHC | 1902.0532_1 | none |
| 1900.0411_1 | CTRPNNNTRKGIYLGSGRAFYTTDKIIGDIRQAHC | 1902.0222_3 | none |
| 1900.0422_1 | CTRPNNNTRKGIYLGPGRAFYTTDKIIGDIRQAHY | 1902.0045_24 | 1917.0242_3 |
| 1900.0442_1 | CTRPNNNTRKGIYLGPGRAFYTTDKIIGDIRQAHC | 1902.0200_4 | none |
| 1900.0444_1 | CTRPNNNTRKGIYLGPGRAFYTTDKIIGDIRRAHC | 1902.0024_43 | 1917.0305_2 |
| 1900.0473_1 | CTRPNNNTRKGIYLGPGRAFSTTDKIIGDIRQAHC | None | none |
| 1900.0481_1 | CIRPNNNTRKGIHFRAREEHFYATDSIIGDIRQAHC | None | none |
| 1900.0488_1 | CIRPNNNTRKGIHFRARGEHFYATDSIIGDIRQAHC | None | none |
| 1900.0491_1 | CIRPNNNTRKGIYLGPGRAFYTTDKIIGDIRQARC | None | none |
| 1900.0495_1 | CTRPNYNTRKGIYLGPGRAFYTTDKIIGDIRQAHC | None | none |
| 1900.0507_1 | CTRPNNNTRKGVYLGPGRAFYTTDKIIGDIRQAHC | 1902.0140_8 | 1917.0650_1 |
| 1900.0529_1 | CTRPNNNTRKGIYLGPGRAFYTTDKIIGDMRQAHC | None | none |
| 1900.0542_1 | CTRPNNNTRRGIYLGPGRAFYTTDKIIGDIRQAHC | None | none |
| 1900.0558_1 | CTRPNNNTRKGIYLGPGRAFYTTDKIIGDIRQAHC | 1902.0174_6 | none |
| 1900.0588_1 | CERPNNNTRQRLSIGPGRSLYTSRRIIGDVKKAHC | None | none |
| 1900.0591_1 | CTRPNNNTRKGIYLGPGGAFYTTDKIIGDIRQAHC | 1902.0034_31 | 1917.0365_2 |

Sequence names were written in the following way, 1900.0001_25988, means subject 19, week 00, .0001 means it was the most common sequence in the set, and 25988 is the number of times it was identically repeated in the 1900 sample.
